# The Bifunctional Dimer Caffeine-Indan Attenuates α-Synuclein Misfolding, Neurodegeneration and Behavioral Deficits after Chronic Stimulation of Adenosine A1 Receptors

**DOI:** 10.3390/ijms25179386

**Published:** 2024-08-29

**Authors:** Elisabet Jakova, Omozojie P. Aigbogun, Mohamed Taha Moutaoufik, Kevin J. H. Allen, Omer Munir, Devin Brown, Changiz Taghibiglou, Mohan Babu, Chris P. Phenix, Ed S. Krol, Francisco S. Cayabyab

**Affiliations:** 1 Department of Surgery, University of Saskatchewan, Saskatoon, SK S7N 5E5, Canada; 2 Department of Chemistry, University of Saskatchewan, Saskatoon, SK S7N 5C9, Canada; 3 Department of Chemistry and Biochemistry, University of Regina, Regina, SK S4S 0A2, Canada; 4 Pharmaceutical and Nutrition Sciences Research Group, University of Saskatchewan, Saskatoon, SK S7N 5E5, Canada; 5 College of Pharmacy and Nutrition, University of Saskatchewan, Saskatoon, SK S7N 5E5, Canada; 6 Department of Anatomy, Physiology, Pharmacology, College of Medicine, University of Saskatchewan, Saskatoon, SK S7N 5E5, Canada

**Keywords:** α-synucleinopathy, adenosine A1 receptor, 1-aminoindan, caffeine, dimer, N^6^-cyclopentyladenosine, neuroprotection, biodistribution, PET-imaging, protein misfolding

## Abstract

We previously found that chronic adenosine A1 receptor stimulation with N^6^-Cyclopentyladenosine increased α-synuclein misfolding and neurodegeneration in a novel α-synucleinopathy model, a hallmark of Parkinson’s disease. Here, we aimed to synthesize a dimer caffeine-indan linked by a 6-carbon chain to cross the blood–brain barrier and tested its ability to bind α-synuclein, reducing misfolding, behavioral abnormalities, and neurodegeneration in our rodent model. Behavioral tests and histological stains assessed neuroprotective effects of the dimer compound. A rapid synthesis of the ^18^F-labeled analogue enabled Positron Emission Tomography and Computed Tomography imaging for biodistribution measurement. Molecular docking analysis showed that the dimer binds to α-synuclein N- and C-termini and the non-amyloid-β-component (NAC) domain, similar to 1-aminoindan, and this binding promotes a neuroprotective α-synuclein “loop” conformation. The dimer also binds to the orthosteric binding site for adenosine within the adenosine A1 receptor. Immunohistochemistry and confocal imaging showed the dimer abolished α-synuclein upregulation and aggregation in the substantia nigra and hippocampus, and the dimer mitigated cognitive deficits, anxiety, despair, and motor abnormalities. The ^18^F-labeled dimer remained stable post-injection and distributed in various organs, notably in the brain, suggesting its potential as a Positron Emission Tomography tracer for α-synuclein and adenosine A1 receptor in Parkinson’s disease therapy.

## 1. Background

The dysregulation of adenosine homeostasis has been linked to aging-related neurodegenerative diseases [1,2]. We recently reported that a 7-day chronic intraperitoneal (i.p.) injection of the adenosine A1 receptor (A1R) agonist N^6^-Cyclopentyladenosine (CPA) produced similar levels of α-synuclein (α-Syn) upregulation and neurodegeneration as in 5-week daily i.p. CPA injections [3,4]. Therefore, the 7-day CPA chronic injection represents a convenient α-synucleinopathy model to test novel compounds with neuroprotective potential and drugs that could increase the CPA-induced neurotoxicity. Some neuroprotective drugs have been found to bind to α-Syn and prevent further aggregation, including caffeine, nicotine, 1-aminoindan and metformin [5]. Conversely, there are other drugs, such as methamphetamine, cocaine, 2-aminoindan and the herbicides paraquat and rotenone that appear to be neurotoxic because they increase α-Syn misfolding and can be correlated with a higher incidence of PD [6,7,8]. Nonetheless, even with drugs such as caffeine or nicotine, there are several problems, including being unsafe at high doses and causing numerous side effects [9]. However, it was argued that linking these compounds and other similar neuroprotective drugs in dimers or trimers would decrease the misfolding and aggregation of α-Syn without risking the safety of the patients [10]. These compounds can also be referred to as bifunctional drugs [11]. They are connected via a six-carbon alkyl linker to minimize solubility issues and retain the flexibility that will allow further binding to α-Syn. Their synthesis and purity have been previously described [10].

The dimers were tested in vitro using techniques such as nanopore analysis, and it was established that they bind to α-Syn and do not cause a compact conformation [10]. Focusing on the caffeine-indan (C_8_–6–I) dimer, the isothermal titration calorimetry results indicate that the dimer has one binding site with α-Syn and a binding constant of 5.3 × 10^4^ M^−1^; thus, it appears to be neuroprotective [10]. Furthermore, the C_8_–6–I dimer was successful in rescuing the one- and two-copied α-Syn-Green Fluorescent Protein (GFP) yeast strain from α-Syn-induced cell death under the control of a galactose promoter. This dimer was also effective in preventing large α-Syn foci in the one- and two-copied α-Syn-GFP yeast [10]. The present in vitro studies of the C_8_–6–I dimer were conducted using molecular docking simulations [12]. This technique allows the prediction of the binding conformation of the eight in vitro α-Syn structures published by Chen’s group [13,14] with our dimer. This technique is helpful in further elucidating the effect of the dimer on the misfolding patterns of α-Syn. Moreover, in the present study, the C_8_–6–I was tested in combination with the A1R agonist CPA. Following 7-day i.p. treatments with vehicle control or with CPA (3 mg/kg) in the absence or presence of C_8_–6–I (3 or 5 mg/kg), the animals underwent a series of behavioral tests, such as Y-maze, open field, and forced swim test. We hypothesized that systemic administration of the dimer would inhibit the CPA-induced upregulation and aggregation of α-Syn similarly to the previously reported neuroprotective effects of the A1R antagonist DPCPX and 1-aminoindan [3,4]. We will use molecular docking to determine whether the dimer interacts with α-Syn to induce a neuroprotective “loop” conformation, which could explain the reduced neurodegeneration and improved behavioral outcomes for animals.

**Figure 1:**
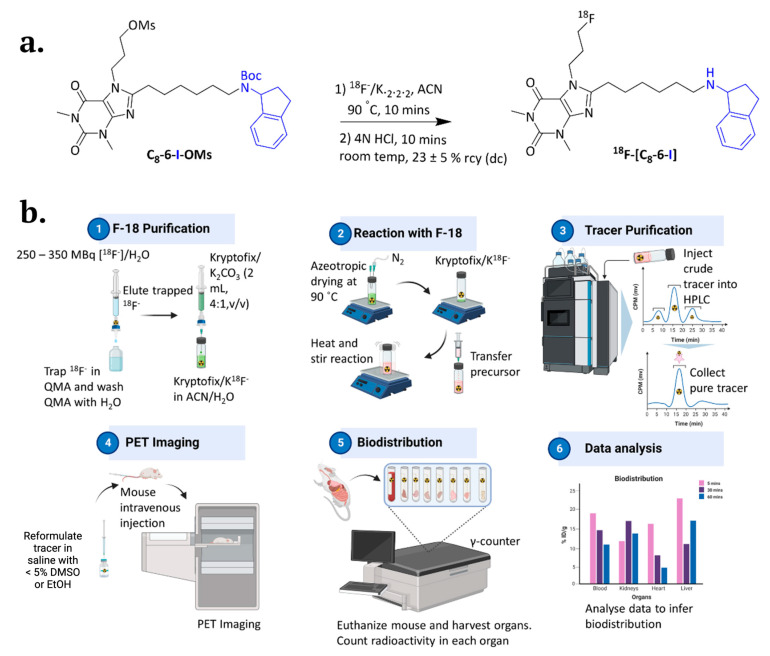
Radiosynthesis of the ^18^F-C_8_–6–I dimer and in vivo studies with the CD-1 mice. (**a**) Formation reaction of ^18^F-C_8_–6–I from C_8_–6–I–OMs in 23 ± 5% rcy (decay corrected). (**b**) All steps involved in the (**b1**) radiosynthesis of ^18^F-C_8_–6–I from [^18^F]fluorine purification, (**b2**) nucleophilic reaction with Kryptofix/K^18^F and (**b3**) semi-preparative HPLC purification, to (**b4**) PET-imaging and (**b5**) biodistribution of the ^18^F- C_8_–6–I in major organs. (**b6**) The data were then analyzed and graphed using GraphPad Prism 8 (San Diego, CA, USA). Created using BioRender.com (URL accessed on 8 July 2024).

To test the blood–brain barrier permeability and the distribution of the dimer, we also performed an in vivo study with CD-1 albino mice using PET imaging. Before PET imaging, the dimer was radiolabeled with fluorine-18 [^18^F] [11]. PET imaging allows the quantitation of drug concentration, distribution, and the pharmacokinetics of radiotracers [15], as well as providing information about physiology such as metabolism, receptor concentration, and transport across cell membranes [16,17]. As a result, PET is a useful tool in drug development [18,19,20]. Since the pharmacological activity of a drug depends on the concentration reaching the tissue of interest, establishing the concentration of free drug reaching organs/tissues of interest and its full biodistribution is an important starting point for early-phase development [17]. PET can be used to non-invasively infer molecule distribution in vivo, as well as brain exposure [17,21]. Recent advances in radiochemistry now mean many drug molecules can be rapidly labeled with radionuclides such as ^11^C or ^18^F, and used for biodistribution studies [22,23]. Radiotracers are commonly used in non-clinical studies to assess the drug absorption, distribution, metabolism and excretion (ADME) profile of a drug [24]. To measure the biodistribution of C_8_–6–I, we have now developed a rapid and robust method for the synthesis of the ^18^F-labeled analogue (as seen in Figure 1) for PET imaging and biodistribution studies. Here, we also report on the radiosynthesis, purification, and in vivo PET imaging and ex vivo biodistribution of ^18^F-C_8_–6–I in healthy CD-1 mice.

## 2. Results

### 2.1. Caffeine-Indan Dimer Decreased the Behavioral Deficits after Chronic A1R Stimulation

**Figure 2:**
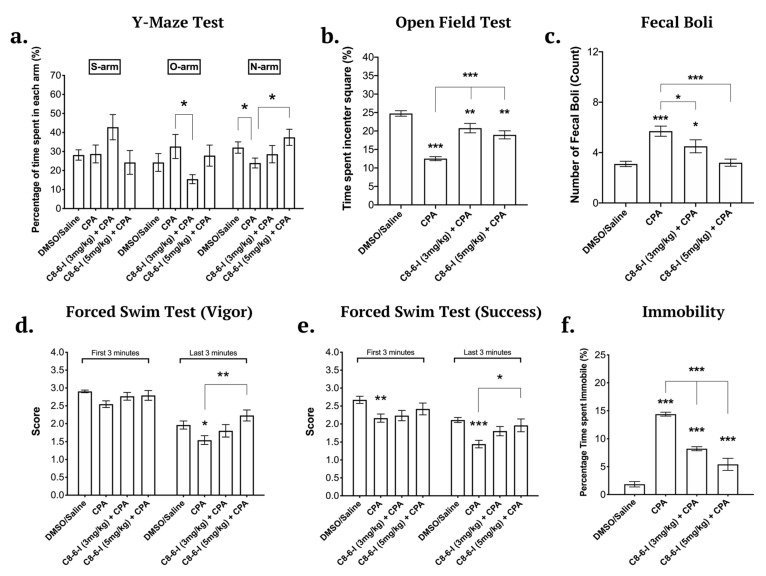
Behavioral tests conducted for male Sprague-Dawley rats after the 7-day chronic injection of C_8_–6–I at 3 and 5 mg/kg. (**a**). Y-maze test values of the 7-day chronic C_8_–6–I dimer (3 mg/kg and 5 mg/kg) as percentage of time spent in each of the arms: S-arm (“start” arm), O-arm (“old” arm), and N-arm (“new’ arm). The percentages of the time spent in each arm were calculated from the 5 min trial. Open field test values of the 7-day chronic C_8_–6–I dimer (3 mg/kg and 5 mg/kg) as percentage of time spent in the center square. The animal is placed in the center square of the grid and left free to explore the field for 10 min. (**b**). The percentage of the time spent in the red center square. (**c**). The total fecal boli count. Forced swim test results of the 7-day chronic C_8_–6–I dimer (3 mg/kg and 5 mg/kg) treatment groups as measurements of swimming vigorously and successfully. The animals were placed in the forced swim tank and let free to swim for 10 min. Once the test was conducted the animals were scored for (**d**) vigor, the ability to purposely swim and use all limbs and (**e**) success, the ability to keep their head above water. (**f**). The total time spent immobile was also measured to assess learned helplessness and despair. Each dimer treatment was repeated at least 10 times per treatment (*n* = 10) and the average of each treatment is presented in bar graphs as means ± SEM. Significances were determined using One-way ANOVA, followed by Student–Newman–Keuls multiple comparison tests with * *p* < 0.05; ** *p* < 0.01; and *** *p* < 0.001.

We assessed the effects of the dimer compound in the recently described α-synucleinopathy model [3,4] involving the daily administration of A1R agonist to Sprague-Dawley rats for 7 days or daily for 5 weeks, and we measured α-Syn expression and aggregation, neurodegeneration and behavioral deficits. Male Sprague-Dawley rats were tested for hippocampal-dependent spatial learning deficits using the Y-maze, for anxiety using the open field test, and for helplessness and motor behavior using the forced swim test [4,25]. The 7-day chronic injections with CPA induced hippocampal-dependent spatial memory deficits that were assessed using the Y-maze test (see Figure 2a). The co-administration of either 3 or 5 mg/kg C_8_–6–I with 3 mg/kg CPA attenuated the learning deficits caused by CPA, as shown by a recovery of the percentage of time spent in the novel arm to closer to the vehicle control values. The 5 mg/kg C_8_–6–I injection significantly ameliorated the cognitive deficits caused by CPA. Another noteworthy observation was the tendency of rats to remain in the start arm when co-administered with CPA + 3 mg/kg C_8_–6–I, as seen in Figure 2a. These rats spent approximately 45% of their time in this arm. A higher percentage of total start arm exploration is a possible sign of anxiety.

The open field test is commonly used to assess anxiety levels [25,26]. During the open field test, the CPA-treated rats spent significantly more time around the perimeter of the square box and less time exploring the center, as shown by the significantly lower percentage of time spent in the center square (Figure 2b). The co-administration of the dimer compound improved the exploratory behavior and reduced the anxiety caused by CPA. Similar to what we previously showed with the A1R antagonist DPCPX improving anxiety, cognitive and motor behavior of rats subjected to 5-week-CPA treatment [4], we now show that both 3 and 5 mg/kg C_8_–6–I doses significantly decreased the fecal boli count, thus indicating that C_8_–6–I attenuated the CPA-induced animal anxiety and improved exploratory behavior (Figure 2b,c).

Initially, when the animals are first placed in water, they swim as vigorously as they possibly can to escape the stressful situation. Often, in the first minute of being in the forced swim tank, animals dive toward the bottom of the tank to explore the corners of the apparatus for a possible exit. However, the time spent underwater exploring is not included in the calculation of vigor or success scores. Nonetheless, towards the end of the forced swim test trial, the animals reach a point of helplessness and fatigue, which is reflected by immobility or “floating” while trying to keep their heads above the water to breathe. The forced swim test showed that CPA induced an increase in motor deficits. However, CPA in the presence of either 3 or 5 mg/kg C_8_–6–I produced no significant motor deficits. However as seen in Figure 2d,e, only the 5 mg/kg bodyweight concentration significantly improved the ability to purposely swim and use all limbs, as well as the ability to keep their head above water. The immobility time calculated in percentage (a measure of helplessness behavior) showed that animals treated with either 3 or 5 mg/kg C_8_–6–I spent significantly less time being immobile compared to the CPA-treated rats (see Figure 2f). This result indicates that C_8_–6–I caused significant improvement of the CPA-dependent depressive-like behavior and motor deficits.

### 2.2. Molecular Docking Revealed Potential Binding of C_8_–6–I to α-Syn and Adenosine Receptors

The α-syn is composed of three functional domains (see Supplementary Figure S1a): the mostly positively charged N-terminus (blue region), the hydrophobic NAC domain (yellow), and the highly disordered negatively charged C-terminus (red). Previous reports using nanopore analysis and molecular docking showed that 1-aminoindan, caffeine, CPA and DPCPX interacted with the different domains of α-Syn [3,6,10]. Using nanopore analysis, previous reports suggest that 1-aminoindan and its derivatives [5,27] as well as the A1R antagonist DPCPX [3] interacted with α-syn in a manner that promotes a neuroprotective “loop” conformation of α-syn. Subsequent molecular docking using the eight soluble α-syn monomeric structures (clusters 1–8, C1–C8) identified by Chen et al. [13] (see Supplementary Figure S1b) revealed that the A1R antagonist DPCPX binds to the α-syn structures C2, C5, C7 and C8, while the 1-aminoindan binds to C1, C4, C5 and C8 structures [3]. Since the dimer compound C_8_–6–I was also previously demonstrated by nanopore analysis to bind to α-syn and prevent α-syn aggregation in a yeast assay [10], we then determined, using molecular docking analysis, if the eight soluble monomeric structures of α-syn interacted with the dimer compound to reveal a neuroprotective “loop” conformation.

**Figure 3:**
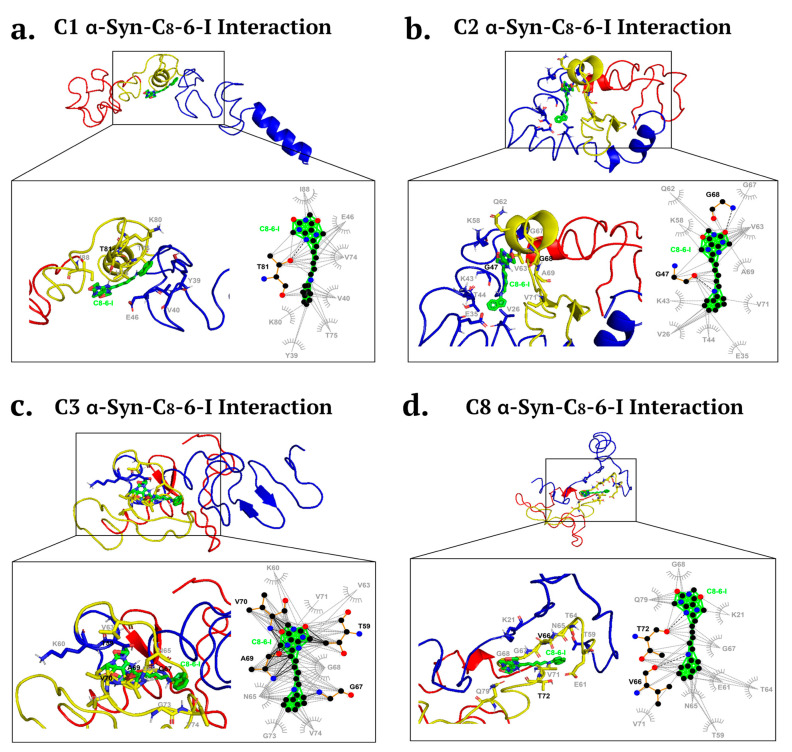
Molecular docking simulation of α-Syn structures ((**a**). C1, (**b**). C2, (**c**). C3, and (**d**). C8) bound to C_8_–6–I. Bold black dashed lines and amino acid residues indicate hydrogen bonding, while the grey dashed lines and amino acid residues indicate hydrophobic interactions. (**a**) The bifunctional dimer compound forms a hydrogen bond with T81 located in the NAC region of α-Syn, and additional hydrophobic interactions are found with the α-Syn N-terminus and NAC region. (**b**) The dimer compound interacts via hydrogen bonding to the α-Syn N-terminus, and additional hydrophobic interactions occur with amino acid residues in the N-terminus and NAC region. (**c**) The dimer compound binds via hydrogen bonds to amino acid residues in the α-Syn N-terminus and NAC region, and additional hydrophobic binding occurs with amino acids located in the distal N-terminus and NAC region. (**d**) The dimer compound binds via hydrogen bonding with NAC amino acid residues (V66, T72) and through hydrophobic interactions with amino acid residues in the N-terminus and NAC domain. C1, C2, C3 and C8 α-Syn structures bind to the dimer compound, which is predicted to form a “loop” conformation of α-Syn.

Therefore, using molecular docking and the same eight α-syn structures (C1–C8) as we previously employed for 1-aminoindan, four α-syn structures, C1, C2, C3 and C8, were selected to analyze the in silico binding of the C_8_–6–I to α-syn. For the C1 structure, C_8_–6–I was shown to form a hydrogen bond with threonine 81 (T81) of the NAC region, as well as undertaking hydrophobic interactions with the hydrophobic side chains of tyrosine 39 (Y39) and valine 40 (V40) and the negatively charged glutamic acid (E46) of the N-terminus (Figure 3a). C_8_–6–I also forms additional hydrophobic interactions with V74, T75, lysine 80 (K80), and isoleucine 88 (I88) in the NAC region. For the C2 structure (Figure 3b), C_8_–6–I was shown to form hydrogen bonds with the glycine 47 and 68 (G47 and G68) in the N-terminus and NAC regions, respectively. C_8_–6–I also forms hydrophobic interactions with the electrically charged cleft of the C2 N-terminus consisting of V26, E35, K43, T44, and K58 amino acids, and with the mostly hydrophobic cleft of the NAC region consisting of glutamine 62 (Q62), V63, G67, alanine 69 (A69), and V71 amino acids. For the C3 structure (Figure 3c), C_8_–6–I was shown to form hydrogen bonds with T59 in the N-terminus and G67, A69, and V70 in the NAC region. In addition, the dimer compound forms weak interactions at the end of the N-terminus and throughout the NAC region with K60, V63, asparagine 65 (N65), G68, V71, phenylalanine 73 (F73), and V74. These three conformations indicate the formation of a “loop” conformation between the N-terminus and farthest NAC region of the protein when the dimer binds to them. However, the C8 structure (Figure 3d) was shown to form different drug–protein complexes that may regulate fibrillation (C8) by only forming hydrogen bonds with the NAC region with V66 and T72, stopping it from misfolding and forming fibril bodies. C_8_–6–I was shown to undergo weak interactions with K21 and T59 in the N-terminus and with Q61, T64, N65, G67, G68, V71, and Q79 in the NAC region. C2, C3 and C8 structures form similar binds with the dimer by engaging both the caffeine and indan moieties of the dimer compound.

**Figure 4:**
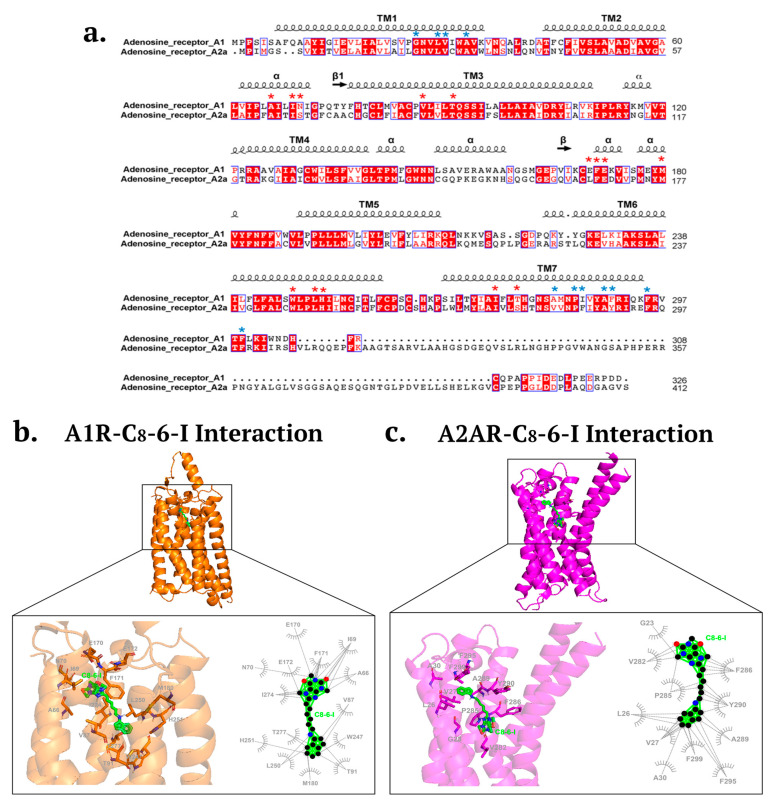
Molecular docking of C_8_–6–I with A1R and A2AR. (**a**) Amino acid sequence alignment of human A1R and A2AR with distinct binding of C_8_–6–I to A1R and A2AR indicated (red asterisks, A1R binding; blue asterisks, A2AR binding). Amino acid residues shaded in red are conserved or identical amino acid sequences, while amino acids in red font are mostly classified under non-polar aliphatic residues (AVLIM). Other amino acids highlighted in red font are classified as follows: HKR are polar positive; DE are polar negative; STNQ are polar neutral; FYW are nonpolar aromatic. (**b**) Molecular docking of C_8_–6–I with A1R showing binding to amino acid residues that are similarly found within the A1R orthosteric binding site for adenosine. (**c**) Molecular docking showing C_8_–6–I binding to amino acid residues that do not resemble those associated with A2AR orthosteric binding site.

Also, since caffeine is a non-selective antagonist of A1R and A2AR, we determined whether the caffeine moiety-containing C_8_–6–I still retained binding activity to A1R or A2AR. Using alpha fold molecular docking of C_8_–6–I to A1R and A2AR, we showed that C_8_–6–I was still capable of binding to and occupying the orthosteric or adenosine-binding pocket of A1R, but not A2AR, as shown in Figure 4. For A1R, the C_8_–6–I binds to similar adenosine orthosteric binding sites, including F171, E172, V87, methionine 180 (M180), L250, tryptophan 247 (W247), T91, T277, and I274 [28]. As shown in Figure 4a,b the C_8_–6–I is shown to bind to the histidine 251 (H251) and leucine 250 (L250) on transmembrane 6 (TM6) and I274 on TM7, which is near the histidine residue (H278 on TM7) [29]. Both H251 and H278 are known to be putatively involved in ligand binding [29]. C_8_–6–I also binds to the second extracellular loop (ECL2) at N70, E170, F171 and E172. C_8_–6–I interacts with amino acid residues on TM3 (V87, T91) and TM7 (I274), which are aligned in the direction of the central cavity [29]. Finally, C_8_–6–I is also shown to bind to the extrahelical lipid-facing pocket and to the residues I69 (TM2), M180 (TM5), W247 (TM6), and T277, which protrude out of the area of the central cavity.

Although the C_8_–6–I undertook hydrophobic interactions with A2AR (see Figure 4a,c), these interactions did not contact amino acid residues that reside within the orthosteric binding pocket of A2AR. The C_8_–6–I formed hydrophobic interactions with residues in TM1 (G23, L26, V27, A30), TM7 (V282, P285, F286, A289, T290), and C-terminus (F299, F295). These residues do not match those found in the orthosteric binding site of A2AR, which binds the agonist adenosine and the naturally occurring antagonist caffeine. The orthosteric binding site for A2AR has been well characterized, and includes amino acid residues that bind both agonists and antagonists (F168, M177, L249, N253, I274) and residues that bind only agonist (T88 in TM3 and S277/H278 in TM7) [30].

Since the dimer C_8_–6–I, the A1R antagonist DPCPX, and 1-aminoindan (a component of the dimer) have been shown by molecular docking and nanopore analysis (present study, and see also [3,10]) to bind to alpha-synuclein, we hypothesized that 1-aminoindan could directly bind to A1Rs, similar to DPCPX and C_8_–6–I, to contribute to neuroprotection during chronic A1R stimulation. Therefore, we also tested whether the 1-aminoindan component of the dimer compound binds to adenosine receptors (A1R and A2AR) (see Supplementary Figure S4a,b). For the A1R, we found that 1-aminoindan forms hydrophobic interactions with the following amino acid residues: F171, L250, I274, I270, and N254 (See Supplementary Figure S4a). In contrast, the C_8_–6–I dimer binds to I274 and F171, but this hydrophobic interaction occurs between the caffeine moiety of the dimer and the I274 and F171 residues of A1R (see Figure 4a,b). For A2AR, we found that 1-aminoindan binds to F163, E164, L244, M265, and I269; these amino acids did not overlap with the C_8_–6–I binding to A2AR (see Figure 4a,c) or with the orthosteric binding site for adenosine or caffeine (see ref. [30]). Therefore, these additional molecular docking data reveal that the monomeric constituents of the dimer (caffeine, 1-aminoindan) have distinct binding sites on A1R and A2AR compared to the dimer compound.

### 2.3. C_8_–6–I Decreased CPA-Induced α-Synuclein Expression and Aggregation in Substantia Nigra and Hippocampus

**Figure 5:**
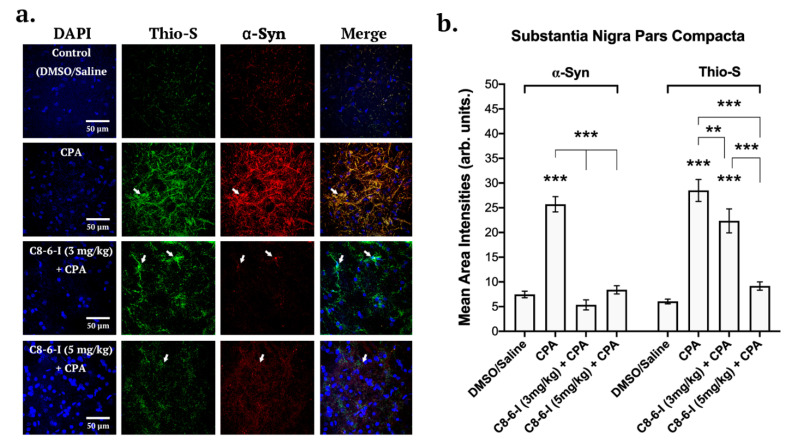
Summary of the surface area analysis of the dimer study’s pars compacta region of the substantia nigra of DAPI, TH, and α-Syn. (**a**) Representative images of 40 μm pars compacta region of substantia nigra taken with 63X oil immersion objective of a confocal microscope (126 times magnification). Separate channels of 7-day chronic intraperitoneal injections with 3 mg/kg of the following treatments: Control (DMSO/Saline), CPA, C_8_–6–I (3 mg/kg) + CPA, and C_8_–6–I (5 mg/kg) + CPA. Slices were probed for DAPI (Blue), Thioflavin S (Thio-S, green), and α-Syn (Red, Alexa Fluor 647). Arrows indicate neuronal somas and processes with high localization of aggregated α-Syn. Scale 50 μm. (**b**) Bar charts showing the mean area intensities of α-Syn and Thioflavin S in the pars compacta region of the substantia nigra. Similar areas of 100-by-100 μm ROI coordinates for lateral pars compacta of SN were quantified, respectively, for each slice and normalized by subtracting F0 (50 by 50 μm ROI coordinates) values of the background (non-cell body bottom area). The average intensity values in bars represent the average mean ± SEM from *n* = 4 independent experiments. Significances were determined using One-way ANOVA, followed by Student–Newman–Keuls multiple comparison tests with ** *p* < 0.01; and *** *p* < 0.001.

**Figure 6:**
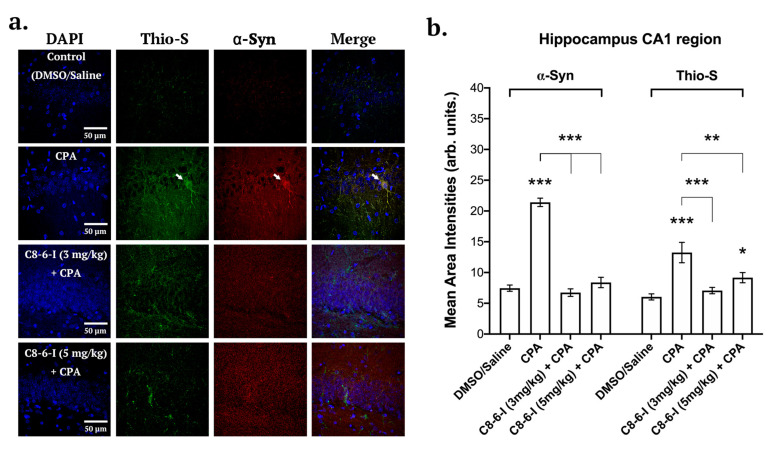
Summary of the surface area analysis of the CA1 region of the hippocampus of DAPI, α-Syn and Thio-S. (**a**) Representative images from 40 μm hippocampal rat brain slices after probing for DAPI, anti-α-Synuclein and Thioflavin S (Thio-S) taken at 63-times magnification with a confocal microscope for the following treatments: Control (DMSO/Saline), CPA, C_8_–6–I (3 mg/kg) + CPA, and C_8_–6–I (5 mg/kg) + CPA. Arrows indicate high colocalization of Thioflavin S and α-Syn in CA1 hippocampal neuronal somas and dendritic processes. Scale 50 μm. (**b**) Bar charts showing the mean area intensities of α-Syn and Thioflavin S in the CA1 region of the hippocampus. Fluorescence intensities from a 100-by-100 μm ROI from the CA1 pyramidal cell layer of the hippocampus were quantified using a similar method to that employed for the pars compacta region. The average intensity values in bars represent the average mean ± SEM from *n* = 4 independent experiments. Significances were determined using One-way ANOVA, followed by Student–Newman–Keuls multiple comparison test with * *p* < 0.05; ** *p* < 0.01; and *** *p* < 0.001.

Brain slices from the 7-day chronic injection model were randomly selected from 6-well plates containing 40 µm coronal brain slices, and fixed hippocampal slices (bregma −3.80 and −4.16 mm) as well SN slices (bregma −5.30 and −5.60 mm) were stained with DAPI, anti-α-Syn and anti-tyrosine hydroxylase (see Figure 5), while the same was performed in parallel slices with DAPI, anti-α-Syn and Thio-S to detect aggregation levels. With C_8_–6–I co-administration with CPA, we found comparable decreased pattern levels of α-Syn in both the SNc and CA1 regions of the hippocampus (Figure 5 and Figure 6). The α-Syn levels in both the SNc and CA1 hippocampal region markedly increased after CPA treatment alone; however, C_8_–6–I co-treatment with CPA prevented this α-Syn upregulation. Interestingly, a significant increase in Thio-S levels was observed in both the CPA-alone treatment and the 3 mg/kg C_8_–6–I + CPA-treated rats compared to control levels in SNc (Figure 5b), while 3 mg/kg C_8_–6–I + CPA treatment abolished the CPA-induced Thio-S levels in the CA1 hippocampus (Figure 6b). However, the Thio-S levels of the 5 mg/kg C_8_–6–I + CPA-treated rats reached control levels in the SNc, while a moderate but significant Thio-S level increase was observed in the hippocampus.

### 2.4. C_8_–6–I Decreased CPA-Induced Neurodegeneration in Substantia Nigra and Hippocampus

**Figure 7:**
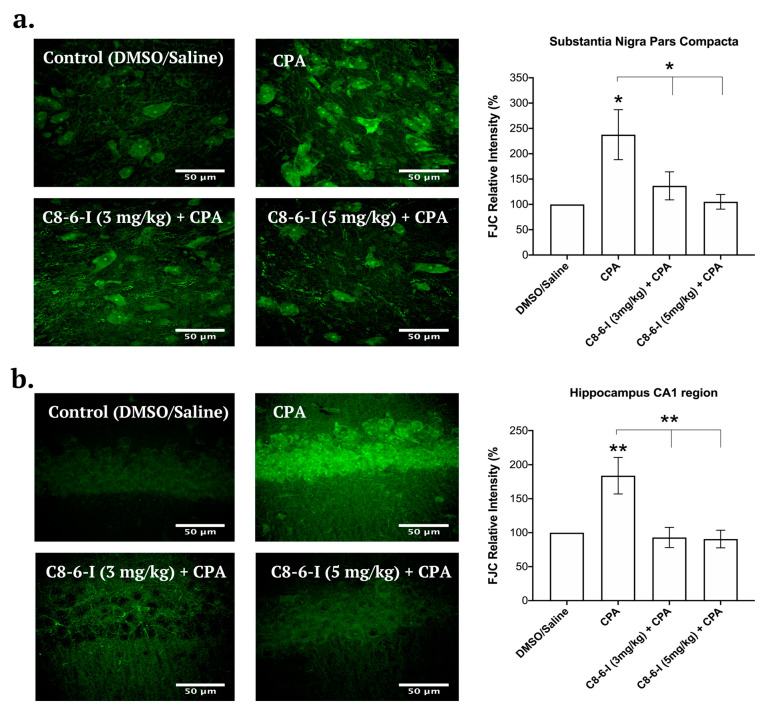
Fluoro-Jade C (FJC) staining in the SN pars compacta and in the hippocampus CA1 region of rats with 7-day chronic intraperitoneal injection of Control (DMSO/saline), CPA, C_8_–6–I (3 mg/kg) + CPA, and C_8_–6–I (5 mg/kg) + CPA. Representative images with 50 μm scale bar for the (**a**) SN pars compacta and (**b**) the CA1 region of the hippocampus. FJC fluorescence intensity in a 100 × 100 μm^2^ region was normalized to the control group (100%). Values are shown as mean ± SEM. The average FJC fluorescence values were obtained from *n* = 4 independent experiments. * *p* < 0.05; and ** *p* < 0.01 (one-way ANOVA followed by Student–Newman–Keuls post-hoc multiple comparison test).

We recently reported that the chronic administration of CPA contributed to increased neuronal damage and the selective inhibition of A1Rs, and the administration of 1-aminoindan both attenuated neurodegeneration and α-Syn aggregation [3]. We then tested C_8_–6–I, which contains both the caffeine and indan moieties, to determine if this dimer exhibited neuroprotective properties in vivo. We used the same FJC protocol [3] as previously established to test for neurodegeneration in the brain. Similar anatomical brain slices to those used in Figure 5 and Figure 6 were utilized in this study (nigral slices −5.30 to −5.60 mm from the bregma as well as hippocampal slices −3.80 to −4.16 mm from the bregma). The SNc representative images indicate that CPA alone increased the levels of FJC fluorescence compared to the control (as summarized in Figure 7a). In contrast, both doses of the C_8_–6–I (3 mg/kg) + CPA and C_8_–6–I (5 mg/kg) + CPA did not significantly increase FJC staining compared to the vehicle control slices. Similar results were observed in the CA1 region of the hippocampus (as summarized in Figure 7b), where treatments with CPA demonstrated much higher levels of degenerating CA1 pyramidal neurons compared to the control, as previously observed [3], or with co-administered C_8_–6–I. Similar to the nigral slices, the co-administration of C_8_–6–I (3 mg/kg) or C_8_–6–I (5 mg/kg) with CPA prevented neurodegeneration in hippocampal slices.

### 2.5. ^18^F-Labeled C_8_–6–I and Its Potential Therapeutic Use for In Vivo Studies

To synthesize the ^18^F-C_8_–6–I, we envisaged that S_N_2 displacement of the mesylated precursor C_8_–6–I–OMs with [^18^F]fluoride [21] would yield the desired radiotracer (as seen in Figure 1). We anticipated that the secondary amine would require protection with tert-butyloxycarbonyl (Boc) to prevent the formation of unwanted byproducts during radiolabeling. Boc could then be rapidly removed using trifluoroacetic acid (TFA) or hydrochloric acid (HCl) under the short reaction times required for ^18^F-radiochemistry. Although the rapid incorporation of ^18^F (acetonitrile, 90 °C) was observed at high yields, as judged by HPLC, N-Boc deprotection with TFA was unsuccessful, with the reaction producing major side products. Despite several attempts to optimize the Boc deprotection step by varying reaction temperature and time, <1% of the radioactive compounds were identified as ^18^F-C_8_–6–I. Alternatively, we decided to use 4 N HCl in dioxane for our N-Boc deprotection at room temperature, and this led to an increase in our total radiochemical yields (rcy) from <1% (using TFA) to 23 ± 5% (*n* = 7, using 4 N HCl), decay corrected, with a radiochemical purity of ≥98% that is suitable for PET imaging in healthy mice.

### 2.6. ^18^F-Labeled C_8_–6–I In Vivo Metabolism and Distribution in the Major Organs Including the Brain

**Figure 8:**
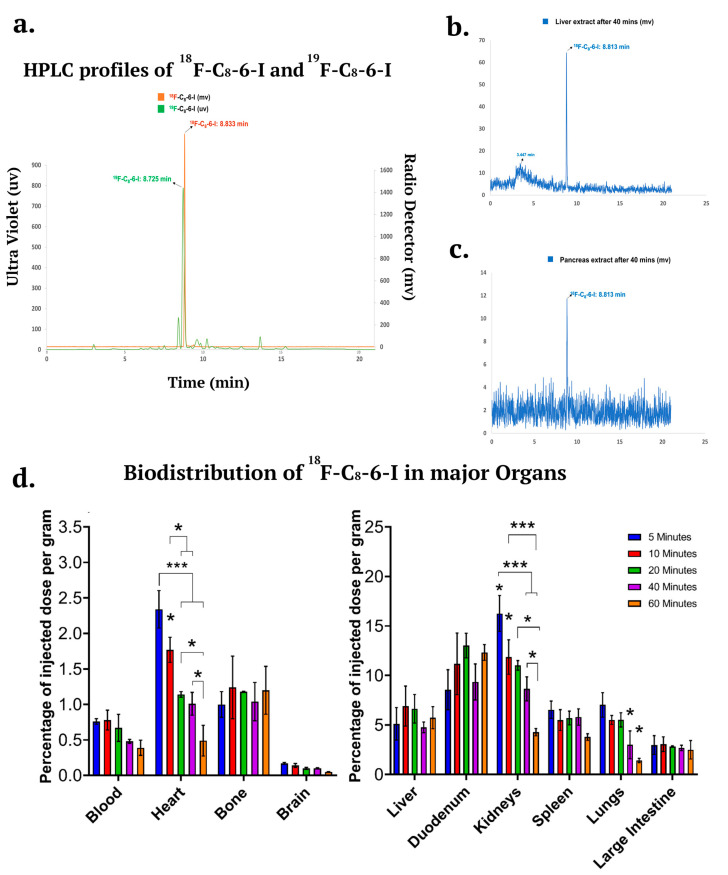
Ex vivo stability and biodistribution in CD-1 mice at five different time points (5, 10, 20, 40, and 60 min) and major organs. (**a**) HPLC co-registration profiles of ^18^F–C_8_–6–I and ^19^F–C_8_–6–I based on a radio detector and ultraviolet detector, respectively. Analytical radio-HPLC chromatograms in mouse (**b**) liver and (**c**) pancreas extracts at 40 min after injection of ^18^F–C_8_–6–I. (**d**) The distribution of ^18^F–C_8_–6–I was calculated as a percentage of the injected dose per gram of tissue (% ID/g) and the results are displayed into two groups: lower distribution—blood, heart, bone, and brain (left panel), and higher distribution—liver, duodenum, kidneys, spleen, lungs, and large intestine (right panel). The data were obtained from *n* = 3 independent animals. Values are presented as mean ± SEM. Significances are indicated as follows: * *p* < 0.05; and *** *p* < 0.001 (one-way ANOVA followed by Student–Newman–Keuls post-hoc multiple comparison test).

To further investigate the pharmacokinetics of ^18^F–C_8_–6–I and its metabolic stability in vivo, a metabolic stability study was carried out via a radio HPLC analysis performed on extracts of liver, pancreas, and brain at 40 min p.i. Previous knowledge of the in vitro metabolic stability profiles of ^19^F–C_8_–6–I and C_8_–6–I in human, mouse and rat liver microsomes [11] suggested that a significant percentage of the parent ^19^F–C_8_–6–I remained after 1 h incubation, which may allow in vivo radio metabolite analysis of ^18^F–C_8_–6–I in various tissues including the brain, liver and GI tract. However, the in vivo metabolic stability of ^18^F–C_8_–6–I has not been analyzed. Therefore, it is crucial to determine whether significant levels of the parent ^18^F–C_8_–6–I remain in the blood plasma and lead to higher uptake in the brain for 1 h to 2 h PET imaging. The radioactivity measured in our studies was mostly related to radioactive emissions from ^18^F–C_8_–6–I (see Figure 8a). Using reverse-phase HPLC with UV detection for ^19^F–C_8_–6–I and radio detection for ^18^F–C_8_–6–I, we determined that the ^18^F–C_8_–6–I was stable with little or fewer metabolites when chromatogram retention times were compared to ^19^F–C_8_–6–I (Figure 8a). Representative radio-HPLC chromatograms from the liver and pancreas (Figure 8b,c) showed that the parent compound ^18^F–C_8_–6–I accounted for ≥95% of the radioactivity signal. We were unable to identify the radioactivity in the brain due to low uptake in the brain and poor sensitivity using radio HPLC. However, the ^18^F–C_8_–6–I was detectable in major organs, including the liver and pancreas, where a single peak was found to correspond to the ^18^F–C_8_–6–I signal around 8.813 min (Figure 8b,c).

^18^F–C_8_–6–I exhibited a moderate CNS multiparameter optimization (MPO) score (4.2), reasonable topological surface area (68.25 Å) and proper lipophilicity (cLogP—3.46 and cLogD—1.26) [31]. However, ^18^F–C_8_–6–I has a high molecular weight (455.57) and high pKa (9.49). To evaluate the pharmacokinetics of ^18^F–C_8_–6–I, whole body ex vivo biodistribution was performed with healthy female CD1 mice at 5, 10, 20, 40, and 60 min p.i. The results of the biodistribution studies are shown in Figure 8d. The data are also summarized in Table 1. Notably, ^18^F–C_8_–6–I displayed low initial brain uptake (0.17 ± 0.014% ID/g) at 5 min p.i. and slow washout over a period of 60 min (0.05 ± 0.004% ID/g), as seen in Figure 8d. The slow clearance of ^18^F–C_8_–6–I from the brain (brain_5min_/brain_20min_ = 1.7, and brain_5min_/brain_60min_ = 3.4) may explain why chronic i.p. injections of C_8_–6–I over the 7 days in the Sprague-Dawley rats not only indicated decreased expression of α-Syn and its aggregates, as seen in Figure 5 and Figure 6, but also significantly lowered the levels of neurodegenerations, as seen in Figure 7. Thus, the dimer has desirable properties as a therapeutic for PD. The bone uptake of ^18^F–C_8_–6–I (≤1.20% ID/g, Figure 8d) was also low, indicating that little defluorination occurred in vivo. The amount of radioactivity in the blood was also low, with initial uptake (0.76 ± 0.04% ID/g) at 5 min p.i. and very slow elimination (blood_5min_/blood_20min_ = 1.1, and blood_5min_/blood_60min_ = 1.9) until 60 min (0.39 ± 0.10% ID/g).

As shown in Figure 8d, several organs, including the kidneys, lungs, spleen, duodenum, and liver, showed high initial uptakes greater than 5% of injected dose per gram (%ID/g) of ^18^F–C_8_–6–I at 5 min p.i.. After high initial uptake, the radioactivity in the kidney, spleen, and lungs displayed a much faster washout than other organs. The kidneys displayed the highest uptake (16.26 ± 1.82% ID/g) of radiotracer at 5 min p.i., with fast elimination over a period of 60 min. The liver showed an uptake of 5.12 ± 1.64% ID/g at 5 min p.i. and 6.91 ± 2.02% ID/g at 10 min p.i. The activity in the liver decreased until 40 min (4.76 ± 0.54% ID/g), before showing an increase again at 60 min (5.74 ± 1.11% ID/g). A similar pharmacokinetic profile can be observed for the duodenum, where activity uptake at 5 min p.i. was 8.56 ± 2.01% ID/g, and this increased until 20 min (13.02 ± 1.23) before decreasing to 9.35 ± 1.81% ID/g at 40 min p.i. However, an increase in uptake (12.33 ± 0.80% ID/g) can be observed at 60 min p.i., similar to that observed for the liver at 60 min (see Figure 8d, right panel). These results of a parallel increase in liver and duodenum at 60 min are consistent with ^18^F–C_8_–6–I undergoing hepatobiliary elimination.

### 2.7. In Vivo PET/CT Imaging in CD-1 Mice

**Figure 9:**
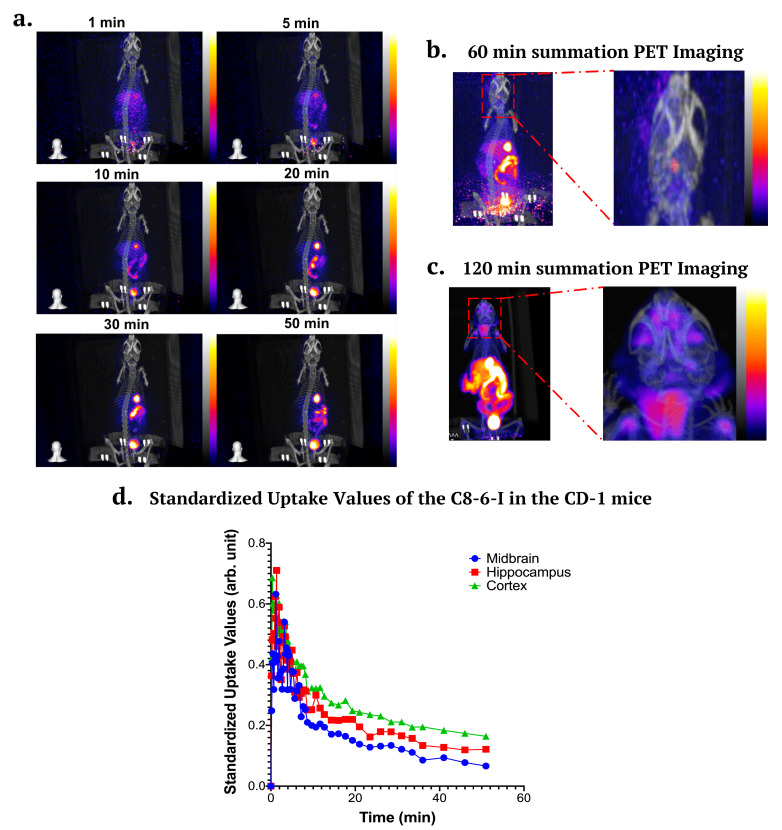
Representative PET/CT images in CD-1 mice at different time points throughout an hour, as well as time–activity curve in the brain. (**a**) PET images at different time points (1 min, 5 min, 10 min, 20 min, 30 min, and 50 min). (**b**) PET summation images for 60 min and (**c**) 120 min dynamic imaging. (**d**) Time–activity curve (TAC) of ^18^F–C_8_–6–I for whole brain and three regions consisting of the cortex, midbrain, and hippocampus from PET/CT imaging. Values are presented as the standardized uptake value (SUV). The data were obtained from *n* = 3 independent animals.

We performed 60 min (*n* = 2) to 120 min dynamic imaging in healthy female CD-1 mice (see Figure 9a–c). Mice were administered with 5.27–5.52 MBq of ^18^F–C_8_–6–I using tail-vein injections. PET dynamic imaging indicates that ^18^F–C_8_–6–I accumulates in the liver, followed by uptake in the gastrointestinal tract. The dynamic image combined with our biodistribution further suggests that ^18^F–C_8_–6–I undergoes hepatobiliary elimination. The 120 PET summation images suggest some uptake in the thyroid and lymph nodes. Neglegible uptake is observed in the bone, which validates that little defluorination occurs for this radiotracer in vivo. However, the image suggests very low brain uptake consistent with our biodistribution studies. The quantification of activity concentration in the brain indicated a maximum of 0.47 standardized uptake value (SUV) in the whole brain, and this slowly decreased to 0.21 after 50 min. The in vivo brain uptake is lower than would be ideal for CNS imaging (peak > 1 SUV); however, the slow decrease from 0.47 to 0.21 SUV after 50 min indicates why the chronic i.p. injection of the dimer makes it suitable for use as a potential therapeutic for PD. In addition to the whole brain, specific anatomical regions were also measured to establish the uptake levels in the cortex, hippocampus and midbrain (see Figure 9d). This demonstration, that ^18^F–C_8_–6–I accumulates in the brain, correlates with the observed in vivo neuroprotective effects of C_8_–6–I (see Figure 7), reduced α-Syn aggregation (Figure 5 and Figure 6), and improved behavioral outcomes (Figure 2) in our 7-day chronic i.p. CPA injection α-synucleinopathy model.

## 3. Discussion

Many epidemiological and multipurpose drug studies have presented interesting observations, such as that the consumption of coffee or tobacco significantly decreased the risk of PD [32,33,34,35,36,37]. Recently, it was shown that caffeine and nicotine are neuroprotective against wild-type and mutant parkin proteins [9]. The substitution of glutamic acid to lysine (E28K) in the ubiquitin-like binding domain of the parkin protein is strongly associated with early onset PD, but also with several cancers such as onset melanoma [38,39]. Caffeine and nicotine were shown to bind directly to the 28th amino acid residue of both parkin and the E28K mutant [9]. These two drugs show neuroprotective effects by decreasing the misfolding of the two proteins. Another interesting finding was the unexpected effects of metformin, a drug used to treat type 2 diabetes, which has been reported to be neuroprotective against PD in long-term studies [40,41,42]. Another study looked at the role of metformin in reducing mitochondrial hyperactivity [43]. The dysfunction of the branched-chain amino acid (BCAA) metabolism pathway was linked to PD-like motor deficits and neurodegeneration. This neurodegeneration was linked directly to hyperactivity of the mitochondria; however, metformin decreased the mitochondria respiration levels. Mitochondria dysfunction has been previously linked with PD in numerous studies [44,45,46]. Moreover, as previously mentioned, 1-aminoindan, a metabolite of rasagiline, is also shown to be neuroprotective [47,48,49]. 1-aminoindan was shown to bind directly to α-Syn, thereby promoting a neuroprotective “loop” conformation that attenuates the α-Syn misfolding and aggregation in a yeast model overexpressing α-Syn, or in an in vivo rodent model of α-Synucleinopathy [3,5,6,27].

Although drugs such as caffeine, nicotine or 1-aminoindan would be clinically relevant for reducing the risk of developing PD, these drugs may be highly toxic at high concentrations [50,51,52]. In addition, there are several side effects of consuming high doses of caffeine or nicotine, such as anxiety and nervousness, fast heart rate, heartburn, high blood pressure, cancer and others [53,54,55]. Therefore, linking these compounds and other similar neuroprotective drugs into dimers or trimers may be a more effective strategy to reduce the misfolding and aggregation of α-Syn without risking the safety of the patients [10,11,56]. These compounds were optimized with a six-carbon alkyl linker, to minimize solubility issues and retain the flexibility that will allow further binding to α-Syn and protect the protein from misfolding and aggregating [10]. We previously reported, using nanopore analysis and isothermal titration calorimetry, that the dimer C_8_–6–I and other caffeine-based dimers (caffeine-nicotine, caffeine-metformin) do indeed bind to α-Syn [10]. Using an in vitro cell death model, we also showed that C_8_–6–I was able to rescue one- and two-copied α-synuclein-Green Fluorescent Protein yeast strains from α-synuclein-induced cell death under the control of a galactose promoter [10]. However, whether this dimer can directly abrogate α-synuclein aggregation in dopaminergic neurons or whether it may promote proteosome clearance of alpha-synuclein aggregates remains to be established. Thus, the present study investigated the role of the C_8_–6–I dimer as a promising neuroprotective agent in our established in vivo 7-day CPA injection α-synucleinopathy model, which displayed increased α-Syn expression, aggregation, and neurodegeneration [3]. The dimer concentration is difficult to attain confidently via conventional approaches, especially for tissue compartments with specialized epithelial or endothelial cell barriers such as the central nervous system (CNS) [17]. For example, the blood–brain barrier (BBB) prevents compounds, typically hydrophilic, from entering the brain, and is a major obstacle that must be overcome during drug development. A lack of adequate brain exposure to drugs often leads to the failure of CNS drug candidates [22,57]. Lipophilic compounds that can passively diffuse across membranes may also be substrates of efflux transporters (such as P-glycoproteins, organic anion transporters, and multidrug-resistance-associated proteins) at the BBB, resulting in the low brain penetration of pharmacologically active drug molecules [22]. To then predict the BBB permeability of ^18^F–C_8_–6–I, we calculated the CNS multiparameter optimization (MPO) desirability score using the compound’s physicochemical properties [58]. For the CNS MPO score, six physicochemical parameters (calculated LogP, calculated LogD, molecular weight, topological polar surface area, hydrogen bond donor, and acidity constant) are normalized and converted to a function ranging from 0 to 1, and the sum of all the normalized physicochemical properties produces a CNS MPO score ranging from 0 to 6. Generally, therapeutic agents with preferable BBB permeability show CNS MPO scores of 4 or higher [59,60,61,62,63,64]. The CNS MPO score for ^18^F–C_8_–6–I was calculated as 4.2. Based on these initial results from binding studies using isothermal titration calorimetry with in vitro yeast assays indicating binding to α-Syn [10], in vitro metabolic studies [11], kinetic measurement and pharmacokinetic predictions [31], and CNS MPO desirability score, we synthesized ^18^F–C_8_–6–I for in vivo evaluation in healthy mice. We found that ^18^F–C_8_–6–I was distributed in the brain regions, including the hippocampus and midbrain, which supports the reduced α-Syn expression and aggregation and neurodegeneration observed in these regions. Although our PET-CT studies showed ^18^F–C_8_–6–I biodistribution and slow elimination in different organs, including the brain, it is as yet unclear whether this PET-tracer compound is binding directly to α-Syn or other targets in the brain to mediate neuroprotection and mitigate behavioral deficits. However, our present molecular docking results and previous reports using nanopore analysis [10] confirm that the C_8_–6–I dimer compound likely binds to α-Syn to promote a neuroprotective “loop” conformation that reduces α-Syn misfolding and aggregation. Moreover, our molecular docking results also indicate that the C_8_–6–I compound could bind to other targets, including adenosine receptors (see below), which further contributes to the observed neuroprotective effects of the dimer in the hippocampus and SN brain regions.

In previous studies, we demonstrated that chronic A1R stimulation with 5 mg/kg CPA for 5 weeks caused a significant increase in α-Syn in the midbrain region, and produced motor and cognitive deficits in male Sprague-Dawley rats [4]. Recently, we also reported a novel 7-day i.p. injection model of 3 mg/kg CPA, indicating similar findings in increased α-Syn expression and aggregation in both the SN pars compacta and the hippocampus [3]. This was demonstrated using confocal images as well as Western blotting techniques. We also showed that chronic CPA treatments increased neurodegeneration levels, as shown by the FJC staining. However, the co-administration of DPCPX or 1-aminoindan prevented the CPA-induced α-Syn aggregation and neurodegeneration [3]. Interestingly, we showed comparable results with the C_8_–6–I dimer. We tested two doses of the dimer, 3 and 5 mg/kg. Following the 7th day of injections, the Sprague-Dawley rats were tested for cognitive dysfunction using the Y-maze test, anxiety levels using the open field test, and kinetic/despair behavior using the forced swim test. We showed that the chronic administration of CPA induced a hippocampal-dependent spatial memory deficit, which was attenuated by co-administration with both doses of the dimer. During the open field test, the CPA group spent significantly less time exploring the center, as shown by the significantly lower percentage of time spent in the center square. The co-administration of the dimer compound improved the exploratory behavior and reduced the anxiety caused by CPA. Moreover, the forced swim test indicated that the CPA-treated rats exhibited reduced vigor and success scores, indicating reduced motor behavior, possibly due to increased despair/helplessness behavior. Both doses of C_8_–6–I used (3 and 5 mg/kg) were effective in restoring cognitive function, anxiety levels and motor activity, and these results are consistent with the observed decrease in neurodegeneration in the hippocampus and SN pars compacta. We did not test the effects of higher doses due to the difficulty in synthesizing and obtaining a high yield of the dimer. However, the initial dose of 3–5 mg/kg C_8_–6–I dimer was sufficient to produce neuroprotective benefits in our α-synucleinopathy model. It is also important to note that the C_8_–6–I dimer is a racemic mixture, and it is not known whether one or both enantiomers are responsible for the activity.

In addition, previous studies with C_8_–6–I and ^19^F–C_8_–6–I indicated that these compounds were promising for use as CNS PET tracers [11,31]. We previously determined that C_8_–6–I directly interacted with α-Syn in vitro and rescued yeast cells from α-Syn fibrillation-mediated toxicity [10]. In the present study, we reported that both the 3 and 5 mg/kg doses of the dimer prevented the CPA-induced α-Syn accumulation and aggregation in both the SN pars compacta and hippocampal CA1 regions. This coincides with the significant reduction in FJC neurodegeneration levels in both the dopaminergic cells of the SN pars compacta and the pyramidal neurons of the CA1 region of the hippocampus. We further synthesized ^19^F–C_8_–6–I, and determined that ^19^F–C_8_–6–I possessed a similar metabolic profile to C_8_–6–I in mouse, rat, and human liver microsomes, and the metabolites are 1-aminoindan hydroxylation, 1-aminoindan *N*-dealkylation, *N*3 and *N*1-demethylation, with the exception of alkyl hydroxylation only found in ^19^F–C_8_–6–I [11]. No defluorination was observed, as this is important for the development of PET probes since fluoride ions can accumulate in the skull and bones, cause toxicity, and confound the interpretation of PET images [11]. To further understand the in vivo parameters of C_8_–6–I and ^19^F–C_8_–6–I, we measured and reported the unbound intrinsic clearance, CL_int_, in mouse liver microsomes, and extrapolated to predict in vivo clearance in mouse plasma, CL_p_, using the well-stirred model. We reported a predicted CL_p_ of 36.0 mL/min/kg for ^19^F–C_8_–6–I compared to 34.3 mL/min/kg for C_8_–6–I [31]. These metabolic stability studies indicate that similar metabolites were observed in mouse, rat and human microsomes, suggesting that mice and rats are appropriate models for future animal studies of ^18^F–C_8_–6–I. Emerging evidence supports the key role of the gut–brain connection in the pathogenesis of PD [65,66,67]. Our pharmacokinetics studies demonstrated a relatively high level of intestinal localization of ^18^F–C_8_–6–I dimer probes, which further suggests the potential applicability of our probe in gut–brain studies.

We also tested whether C_8_–6–I could act on other targets, including A1Rs and A2ARs, since this bifunctional compound contains the caffeine moiety, which could retain its binding affinity for adenosine receptors. Whereas caffeine non-selectively binds to the respective orthosteric adenosine binding sites in A1Rs and A2ARs [29,30], the C_8_–6–I dimer appears to bind preferentially to the A1R orthosteric site. Using molecular docking, we found that C_8_–6–I interacted with the A1R orthosteric binding site, and additional putative allosteric binding sites were also identified. In contrast, C_8_–6–I did not make similar contacts with the orthosteric binding site or allosteric binding site for A2AR. Important amino acid residues in the second extracellular loop (ECL2) of A1R have been identified to play a crucial role in A1R allosteric modulation. Previous studies employing an all-atom Gaussian accelerated molecular dynamics (GaMD) simulation using an inactive A1R structure (PDP: 5UEN) supported the key role of residue E172 (in the ECL2 region) as an important binding determinant for the two positive allosteric modulators (PAMs), PD71723 and VCP171. The alanine substitution of E172 decreased the binding affinity of two allosteric modulators, PD81723 and VCP171 [68,69]. The residues F171 (also in ECL2) and N254 on TM6 are known to interact with orthosteric antagonists DU172 and PSB36 [68], as well as the endogenous agonist adenosine (PDB:6D9H) [28].

In the present study, we observed the binding of C_8_–6–I to the PAM-binding residue E172 and the orthosteric agonist/antagonist binding residue F171. Since we found that C_8_–6–I was effective in preventing the neurodegeneration of hippocampal and substantia nigral neurons, which likely depended on preserving A1R expression, this suggests that C_8_–6–I may act as an A1R PAM that promotes less A1R desensitization compared to A1R orthosteric agonists, adenosine and CPA. However, the C_8_–6–I binding to the orthosteric site F171 also suggests that C_8_–6–I may reduce agonist binding and A1R activity. Although the influence of C_8_–6–I allosteric binding on A1R binding and function has yet to be investigated, it is also possible that the allosteric mechanism may involve decreasing the rate of orthosteric agonist dissociation and enhancing agonist binding and function [70]. This potential A1R allosteric ligand property of C_8_–6–I may be therapeutically beneficial and warrants further studies due to potential therapeutic implications. If C_8_–6–I is an allosteric modulator of A1R, it will be important to carry out further concentration–response analyses, as some A1R PAMs have been shown to act as inhibitors at high concentrations and cause a functional response in the absence of an agonist (e.g., T62), while some compounds (e.g., PD81723 derivative with bridged 3- and 4-positions) exhibited A1R antagonism at low concentrations, but PAM activity at higher concentrations [68,71].

Future functional and biochemical studies are needed to further assess the influence of C_8_–6–I binding to A1R and A2AR in promoting neuroprotection. For example, to test the possibility that dimer binding antagonizes A1Rs, performing electrophysiological recordings of field excitatory post-synaptic potentials (fEPSPs) in rat hippocampus would be a convenient assay for A1R function [72]. If the dimer binds to and antagonizes the A1Rs, then the fEPSP amplitudes are expected to increase, which is consistent with the attenuation of A1R-mediated inhibition of transmitter release. Notably, we also found that the dimer is predicted to bind to the agonist/antagonist orthosteric binding site F171 of A1R. Therefore, future additional radioligand binding studies using ^3^H-DPCPX are planned to confirm whether the C_8_–6–I dimer binds to A1R with affinity of less than 100 nM (similar to the affinity of adenosine), making this dimer an A1R ligand. Moreover, future biochemical analyses and surface biotinylation of surface-expressed A1Rs will help elucidate whether the dimer binding to the extracellular loop 2 (ECL2) allosteric binding site (E172) leads to greater surface expression of the A1R (reflecting decreased receptor desensitization). In addition, via in silico modeling, we also showed the binding of C_8_–6–I to A2AR, but this binding does not appear to be near the orthosteric adenosine binding site of A2AR. Therefore, it is possible that C_8_–6–I may bind not only A1R (either as an antagonist or as a positive allosteric modulator) to mediate neuroprotection, but also A2AR to mediate an as yet unknown A2AR-specific neuroprotective mechanism, which warrants validation in future studies.

In summary, we showed further evidence that a 7-day chronic CPA i.p. injection rat model generated increased α-synuclein expression and aggregation, which provides a convenient model to assay several neuroprotective drugs, including the bifunctional compound C_8_–6–I, without waiting several months to observe the presence of α-synucleinopathies normally seen in genetic mouse of models of PD (e.g., A53T mouse model). Our results indicate that the C_8_–6–I dimer compound promotes neuroprotection by decreasing the CPA-induced α-synucleinopathy and neurodegeneration in the hippocampus and SN pars compacta. This likely involves C_8_–6–I directly binding to α-Syn domains that promote the neuroprotective “loop” conformation, which prevents α-Syn misfolding and aggregation. Moreover, directly linking caffeine to 1-aminoindan also appears to increase the caffeine moiety’s preferential binding to the A1R adenosine orthosteric binding site. Therefore, the C_8_–6–I dimer likely includes, but not exclusively, two direct actions as follows: (1) direct binding to alpha-synuclein to prevent misfolding; and (2) direct binding to the A1 receptor to inhibit the CPA-induced upregulation of alpha-syn. On the one hand, our molecular docking analysis and previous nanopore analysis along with the in vitro yeast cell death assay provide support for our current findings that the dimer inhibits α-Syn aggregation in vivo, all consistent with a putative direct action of the dimer on α-Syn. However, future in vitro studies are needed to determine whether the incubation of α-Syn monomers with the dimer can directly prevent or reverse oligomer formation. On the other hand, our molecular docking study also predicted that the dimer directly interacts with the A1R adenosine orthosteric binding site, which is the same site as that occupied by the non-selective A1R antagonist caffeine and the selective A1R antagonist DPCPX [29]. Moreover, we showed in the present study that the C_8_–6–I dimer prevented CPA-induced alpha-synuclein upregulation, aggregation and neurodegeneration, similar to the effects previously observed with DPCPX [3]. Finally, the C_8_–6–I dimer significantly attenuated the behavioral deficits produced by CPA (present study), which is identical to the inhibitory actions of the A1R antagonist DPCPX and the dimer constituent 1-aminoindan on CPA-induced behavioral abnormalities (Jakova and Cayabyab, personal communication). Therefore, we conclude that the C_8_–6–I dimer actions in the brain are consistent with a direct binding to alpha-synuclein and A1Rs, which contribute to the observed neuroprotection reported in this study. This implies that the observed biodistribution of C_8_–6–I dimer inside the CNS makes it an ideal therapeutic for PD, as this bifunctional drug promotes neuroprotection by inhibiting α-Syn misfolding and aggregation and by suppressing chronic adenosine A1 receptor stimulation, which is expected to prevent α-Syn over-expression. Finally, this study indicates that C_8_–6–I may be an appropriate PET-CT tracer that can be used for imaging diagnostics and the treatment of PD.

## 4. Methods

### 4.1. In Vivo Animal Protocol

Our in vivo studies were designed to test the effects of C_8_–6–I on α-Syn aggregation and neurodegeneration induced by 7 days of i.p. injections of CPA (3 mg/kg) in male Sprague-Dawley rats (28–30 days old, 250–300 g), which were then assessed by behavioral and histological experiments. To test for the biodistribution and pharmacokinetics of radiolabeled C_8_–6–I, female Swiss Albino CD-1 mice (34–46 g) were used. All animals were housed and treated humanely following the guidelines from the following governing bodies: National Research Council (US) Committee for the Update of the Guide for the Care and Use of Laboratory Animals (Washington DC, USA, 2011); Canadian Council on Animal Care (CCAC); and the University of Saskatchewan Animal Research Ethics Board (AREB), which approved our Animal Use Protocol (#20070090 and #20170079). The animals were housed in cages of two, with access to food pellets and water ad libitum. The four treatments of the Sprague-Dawley rats consisted of the following: 1. Control (0.1% DMSO in 0.9% saline); 2. CPA; 3. C_8_–6–I (3 mg/kg) + CPA, and 4. C_8_–6–I (5 mg/kg) + CPA. Drugs were dissolved at 3 mg/mL in DMSO, and each drug was administered to the animals by daily i.p. injections (3 mg/kg or 5 mg/kg body weight) for 7 consecutive days. After the first injections with C_8_–6–I 3 mg/kg or C_8_–6–I 5 mg/kg, the animals were returned to their cages for 30 min before a subsequent CPA injection was administered. Then, on days 8 and 9 following the final injections, the animals were subjected to several behavioral tests.

### 4.2. Reagents

CPA was purchased from Abcam (Toronto, ON, Canada), and DPCPX from Tocris (Burlington, ON, Canada). Formic acid (LC-MS grade purity), 4 N HCl in dioxane, and Kryptofix 222 were purchased from Sigma Aldrich (Oakville, ON, Canada), anhydrous acetonitrile from Thermo Fisher Scientific (Waltham, MA, USA), and sodium bicarbonate from EM SCIENCE (affiliate of Merck, KGaA, Darmstadt, Germany). As seen in Figure 1(b1), [^18^F]Fluoride was produced using the ^18^O(p,n)^18^F reaction with 24 MeV protons in an Advanced Cyclotron System Inc (ACSI) high-current TR-24 cyclotron at Saskatchewan Centre for Cyclotron Sciences (Saskatoon, SK, Canada). The C_8_–6–I–OMs precursor was synthesized according to our previously described synthetic procedure [11].

### 4.3. Behavior Tests

#### 4.3.1. Y-Maze

The Y-maze or the modified Y-maze is a rodent behavior test used to assess short-term spatial memory based on the innate inclination of animals to explore new areas not previously seen [4]. In this study, the Y-maze test is used to assess short-term memory, especially hippocampal-dependent spatial memory [25,26]. The maze is shaped like a “Y”, with each arm oriented at a 120° angle from one another. The arms are 50 cm in length, 15 cm in width and 35 cm in depth. For the first trial, which lasts 15 min, the animal is placed at the beginning of the “Start arm” and is free to explore this arm and another one (referred to as the “Old arm”), while the remaining arm (“New arm”) is blocked. This trial should give the animal time to explore and become acquainted with the maze’s two arms. After a resting period of 90 min, the blocked arm is opened, and the animal is placed again at the “Start arm”. The animal is free to explore all arms including the “New arm” for 5 min. This trial is then used to calculate the percentage of time the animals spent in each arm. A video camera was used to record the animal movement in both trials. Each experiment was repeated at least fifteen times per treatment and the average of each treatment is presented in the bar graphs as average means ± standard error of the mean (SEM).

#### 4.3.2. Open Field Test

The open field is a general locomotor test that detects anxiety and willingness to explore [25,26]. The test is conducted in a white open cube measuring 50 cm in length, 50 cm in width and 35 cm in depth. The bottom field is gridded in 4x4 squares at 12.5 cm, with a center square in the middle of the field. The animal is placed in the center square and is free to explore the entirety of the field for 15 min. The first 5 min are considered an acclimation to the maze, and therefore are not calculated as part of the behavior test. This trial is then used to calculate the percentage of time the animals spent in the center zone (red square) and the total fecal boli count. A video camera was used to record the animal movement in this test. The open field test was conducted after a 1 h resting period from the time the Y-maze second trial was completed. Each experiment was repeated at least ten times per treatment and the average of each treatment is presented in the bar graphs as average means ± SEM.

#### 4.3.3. Forced Swim Test

The forced swim test is used to assess depressive behavior such as despair and learned helplessness [4]. The test is also used to assess the vigor and the ability of the animal to keep its head above the water in which the animals swim [73,74]. In addition, the first and last three minutes of the test were manually scored for the ability of the rat to swim with all its four limbs. Vigor: swim with four limbs—Score 3; occasionally float—Score 2.5; floating more than swimming—Score 2; occasionally swimming—Score 1.5; occasionally swimming with hind limbs—Score 1; no use of limbs—Score 0. In addition, the ability of the animal to keep its head above water was scored. Success: entire head above water—Score 3; ears below water—Score 2.5; eyes below water—Score 2; entire head below water for 3 s—Score 1.5; entire head below water for 6 s—Score 1; in the bottom of tank for more than 10 s—Score 0.

The forced swim test apparatus was a plexiglass cuboid measuring 30 cm in length, 30 cm in width and 60 cm in depth. The tank was then filled two-thirds with lukewarm water not exceeding 25 °C. Afterwards, the animal was left in the cylinder for 10 min during which its time spent immobile as well as its ability to swim and keep its head above water was analyzed. This trial was then used to calculate the percentage of time the animals spent immobile, as well as the vigor and success with which the animal swam. A video camera was used to record the animal movement in this test. The forced swim test was conducted the following day (day nine), giving the animals a sufficient resting period from the Y-maze and open field tests. Each experiment was repeated at least fifteen times per treatment, and the average of each treatment is presented in the bar graphs as average means ± SEM.

### 4.4. Structural Modeling and Molecular Docking

The structures of soluble α-Syn monomers (C1–C8, see Supplementary Figure S1B) have previously been identified and were recently applied to predict the binding of A1R ligands and 1-aminoindan to α-Syn [13]. The C1–C8 α-Syn structures were used in our molecular docking simulations to predict the dimer–protein complexes, and these structures were obtained from PDB-DEV (Entry: PDBDEV_00000082) [14]. The molecular docking study was carried out using the Autodock Vina module implemented in the PyRx tool [3,12]. Protein and ligand interactions were analyzed and visualized through Pymol and LigPlot+.

### 4.5. Immunohistochemistry

Anesthetized rats were transcardially perfused with 0.9% saline, and then fixed with 4% paraformaldehyde. The extracted brains were put in 30% cryoprotected sucrose solution for 48 h prior to slicing. The brains were initially frozen at −40 °C (BFS-30 mp controllers) and sliced with the help of a microtome (Leica SM2010 R Sliding controller). Coronal slices of 40 μm were then washed three times in 0.1 M phosphate-buffered saline followed by 1 h blocking at room temperature with blocking buffer. The buffer solution components have been previously described [74]. The slices were then incubated overnight at 4 °C with the following primary antibodies: 1:200 mouse monoclonal to α-synuclein (Abcam Inc, Toronto, ON, Canada; Cat# ab280377) and 1:200 rabbit polyclonal to tyrosine hydroxylase (TH) (Millipore-Sigma, Oakville, ON, Canada; Cat# AB152). Subsequently, slices were then incubated for 1 h in the dark at room temperature with the following secondary antibodies (1:1000): AlexaFluor-555-conjugated anti-mouse (Cat# A-21127) and AlexaFluor-647-conjugated anti-rabbit (Cat# A-21244) purchased from Invitrogen (Thermo Fisher Scientific, Waltham, MA, USA). Slices were then treated with Thioflavin-S (see further details below). Lastly, the slices were incubated for 5 min at room temperature with DAPI (2 mg/mL) from Invitrogen (Thermo Fisher Scientific; Cat# R37606) and images were taken using a Zeiss LSM700 confocal microscope (Carl Zeiss Group, Toronto, ON, Canada) and analyzed with ImageJ (Open-Source Public Domain software, United States National Institute of Health (US NIH), Bethesda, MD, USA). Images of the hippocampal CA1 pyramidal layer and the SN pars compacta were obtained using the Zeiss Plan-Apochromat 63X/1.4 oil objective lens (Carl Zeiss Group, Toronto, ON, Canada). Images were acquired as Z-stack images of hippocampal or SN regions with 12–13 Z-stack images taken at 1 µm intervals near the middles of brain slices. Two Z-stack images were taken along the hippocampal CA1 or SN pars compacta region for each slice, and immunofluorescence signals were averaged using densitometry analysis.

### 4.6. Thioflavin-S

Thioflavin-S (Thio-S) is a fluorescent marker (Sigma-Aldrich, Oakville, ON, Canada; Cat# T1892-25G) that detects α-Syn aggregates and amyloid plaques. Coronal slices of 40 μm were firstly treated with 0.3% KMnO_4_ for 4 min, followed by a 30 min incubation with 1 M phosphate buffered saline at 4 °C. These slices were then stained with 0.05% Thioflavin-S in 50% ethanol in the dark for 8 min, rinsed with 80% ethanol twice, followed by three rinses with ultra-pure water for 30 s. Finally, the slices were incubated again with 1 M phosphate buffered saline for 30 min at 4 °C before starting the DAPI stain. The FITC filter (488 nm laser line) was used to image Thioflavin-S using a Zeiss LSM700 confocal microscope (Carl Zeiss Group, Canada) and images were analyzed with ImageJ (Public Domain).

### 4.7. Fluoro-Jade C

Fluro-Jade C (FJC) is a fluorescent marker for neurodegeneration (Millipore-Sigma, Oakville, ON, Canada; Cat# AG325). Coronal slices of 40 μm were mounted on 5% gelatin-coated super-frost plus microscope slides (Thermo Fisher Scientific, Waltham, MA, USA) and dried overnight at 4 °C. Initially, the microscope slides were immersed in 1% NaOH/80% ethanol for 5 min followed by 2 min immersion in 70% ethanol. The slides were then rinsed for 2 min with ultra-pure water. The microscope slides were further immersed in 0.06% KMnO_4_ for 10 min, followed by an additional rinse for 2 min with ultra-pure water. The slides were then stained with 0.004% FJC in 0.1% acetic acid for 20 min with gentle shaking on an orbital shaker. Lastly, the slides were rinsed three times in ultra-pure water for 1 min each, making sure to remove all the excess water after each rinse. The slides were then rinsed in xylene and allowed to dry overnight at 4 °C. Then they were treated with Prolong Gold Antifade Reagent from Invitrogen (Thermo Fisher Scientific, Waltham, MA, USA), and respective images were taken using a Zeiss LSM700 confocal microscope (Carl Zeiss Group, Canada) and analyzed with ImageJ (Public Domain). FJC fluorescence was obtained by exciting the dye with a 488 nm laser.

### 4.8. Radiochemistry

^18^F–C_8_–6–I was synthesized from the corresponding mesylate precursor, C_8_–6–I–Oms, using a direct S_N_2 displacement with [^18^F]fluoride (see Figure 1a). [^18^F]Fluoride was trapped on a quaternary methyl ammonium (QMA) cartridge purchased from Waters (Waters, Milford, MA, USA) and eluted off with 2 mL of Kryptofix 2.2.2./K_2_CO_3_ solution (16.0 mg of Kryptofix 2.2.2 and 3.45 mg of K_2_CO_3_ in 2 mL acetonitrile/water, 4/1, *v/v*) into a clean glass vial (see Figure 1(b2)). The solvent was then removed at 90 °C under a stream of nitrogen gas, and the residue was azeotropically dried with anhydrous acetonitrile (2 × 1 mL) at 90 °C. The vessel was cooled to room temperature, and a solution of the mesylate precursor, C_8_–6–I–OMs (1.5–2.0 mg) in anhydrous acetonitrile (0.5 mL), was added. The reaction mixture was heated to 90 °C and stirred for 10 min. The vessel was then cooled again to room temperature, and 4 N HCl in dioxane (0.25 mL) was added. The reaction mixture was stirred at room temperature for another 10 min (see Figure 1(b2)). Saturated NaHCO_3_ solution (0.55 mL) was added slowly to quench the reaction, followed by the purification of the reaction mixture with Ultra-High-Performance Liquid Chromatography (UHPLC) (Thermo iFisher, Waltham, MA, USA) (see Figure 1(b3)).

HPLC purification and analyses of the radiotracer were performed on a Thermo Fisher Vanquish UHPLC system equipped with a Chromeleon 7 communication software version 2.1 (Thermo Fisher, Waltham, MA, USA), Thermo/DIONEX UltiMate AFC-3000 fraction collector, an Agilent Zorbax Eclipse XDB-C18 Semi Prep Column (5 μm C18, 9.4 × 250 mm reverse phase column, PN-990967-202, flow rate 5.00 mL/min), and an Eckert & Ziegler flow-count radio-HPLC detection system. To purify ^18^F–C_8_–6–I, the reaction mixtures were separated on UHPLC using gradient elution with a binary solvent system as described in Table 2. A sample of pure ^19^F–C_8_–6–I was used as an authentic standard to determine the expected retention times of ^18^F–C_8_–6–I. Compound elution was monitored using both absorbance at 280 nm and radiodetection (20k max counts per minute (cpm) and 5 s integration time) with all chromatography done at room temperature. The purified fractions containing ^18^F–C_8_–6–I were rapidly evaporated using the V-10 solvent evaporator (Biotage, Uppsala, Sweden) at 36 °C, and the concentrate was reconstituted in sterile saline solution containing 5% DMSO for animal studies. The total synthesis time was about 0.5 h. The radiochemical yield (rcy) was 23 ± 5% (*n* = 7, decay corrected), and the radiochemical purity was ≥98%, as judged by UHPLC.

### 4.9. Micro-PET/CT Imaging

Female CD-1 mice (34–46 g) were anesthetized with isoflurane (1.5–2.5%) in oxygen at a flow rate of 1–2 L/min and the tail vein was injected with ^18^F–C_8_–6–I (5.27–5.52 MBq, 100 μL) in sterile saline containing <5% DMSO (see Figure 1(b4)). MicroPET dynamic imaging was acquired on a Sofie GNEXT PET/CT scanner following the injection of ^18^F–C_8_–6–I. The field of view (FOV) was 12 cm transaxial and 10.4 cm axial with a spatial resolution of <1 mm. The acquisition of dynamic PET data (10 × 10, 8 × 15, 6 × 20, 6 × 30, 4 × 60, 1 × 100, 4 × 100, 6 × 150, 4 × 300) was started immediately after radiotracer injection, followed by a 1 min CT scan. PET/CT images were registered and reconstructed automatically by the GNEXT system. PET images were reconstructed using a 3D-ordered Subset Expectation Maximization algorithm with 24 subsets and 3 iterations. CT images were reconstructed using a Modified Feldkamp algorithm. All data were analyzed using Vivoquant software (IviCRO) (InVicro, Needham, MA, USA). CT images were used to define anatomical regions of interest (ROIs) through PET/CT image co-registration. The quantification of activity concentration in the brain was performed by drawing ROIs and the mean and maximum standard uptake values (SUVs) were determined using the formula: SUV = [(MBq/mL) × (animal wt. (g))/injected dose (MBq)].

### 4.10. In Vivo Metabolic Stability and Biodistribution

In vivo, the metabolic stability of ^18^F–C_8_–6–I was assessed following a protocol by Zhang and colleagues [75]. After a tail vein injection of ^18^F–C_8_–6–I, the mice were euthanized (anesthesia followed by cervical dislocation) at 40 min post-injection (p.i.) (see Figure 1(b5)). The liver, pancreas, and brain were collected, washed with saline, placed in water/acetonitrile, and homogenized using the Fisherbrand™ 150 Handheld Homogenizer (Thermo Fisher Scientific, Waltham, MA, USA). The samples were centrifuged at 14,000 relative centrifugal force (rcf) for 5 min (AccuSpin Micro 17, Thermo Fisher Scientific, Waltham, MA, USA). The supernatants were collected and filtered using 0.22 μm organic Millipore-Sigma filters (Oakville, ON, Canada). The clarified filtrate was injected into the HPLC and radioanalytes separated by a reverse phase column (5 μm C18, 9.4 × 250 mm reverse phase column, PN-990967-202, flow rate 5.00 mL/min) using the same gradient elution as described in Table 1. The elution of radioactive metabolites was detected using the radiation detector as described above. As seen in Figure 1(b5), biodistribution was performed in healthy female CD-1 mice (54 ± 5.2 g). The purified ^18^F–C_8_–6–I (about 400 kBq–700 kBq/100 μL) in sterile saline containing <5% DMSO was injected into the mice (five groups, *n* = 3 for each group) via the tail vein. The mice were euthanized (anesthesia followed by cervical dislocation followed by cardiac puncture and blood draw) at 5, 10, 20, 40, and 60 min p.i., and different organs were harvested (blood, liver, small intestine, kidney, spleen, lung, heart, large intestine, bone, and brain), washed and placed in pre-weighed tubes. The radioactivity in each organ/tissue was measured using a calibrated automatic gamma counter (2480 Perkin Elmer, Waltham MA, USA) and the tissue uptake was expressed as a percentage of injected dose (ID) per gram (% ID/g). All radioactivity measurements were corrected for decay. Data are shown as average mean± SEM, calculated using replication for each sample.

### 4.11. Statistical Analyses

For behavioral studies, histological, radiotracer biodistribution and Western blot analyses, as well as statistical analyses, were conducted with GraphPad Prism 8 software (GraphPad Software Inc., La Jolla, CA, USA) with one-way ANOVA followed by Student–Neuman–Keuls multiple comparison post hoc tests. The significances are indicated as ns, non-significant; * *p* < 0.05; ** *p* < 0.01; and *** *p* < 0.001.

## Figures and Tables

**Table 1:** Biodistribution of ^18^F–C_8_–6–I in healthy CD-1 mice. Data shown are percentage of injected dose per gram of tissue (%ID/g). The values are presented as mean ± SEM, *n* = 3.

Organ	5 min	10 min	20 min	40 min	60 min
Blood	0.76 ± 0.040	0.78 ± 0.14	0.67 ± 0.19	0.48 ± 0.029	0.39 ± 0.11
Liver	5.12 ± 1.65	6.91 ± 2.02	6.64 ± 1.44	4.76 ± 0.54	5.74 ± 1.12
Small intestine	8.56 ± 2.02	11.18 ± 3.11	13.02 ± 1.24	9.35 ± 1.82	12.33 ± 0.81
Kidney	16.26 ± 1.82	11.86 ± 1.74	11.02 ± 0.48	8.65 ± 1.21	4.27 ± 0.37
Spleen	6.54 ± 0.88	5.49 ± 1.05	5.71 ± 0.69	5.80 ± 0.83	3.79 ± 0.33
Lung	7.03 ± 1.24	5.51 ± 0.44	5.52 ± 0.72	3.00 ± 1.40	1.42 ± 0.22
Heart	2.34 ± 0.26	1.77 ± 0.17	1.14 ± 0.040	1.01 ± 0.16	0.49 ± 0.018
Large intestine	2.98 ± 0.94	3.06 ± 0.73	2.83 ± 0.068	2.69 ± 0.26	2.50 ± 0.93
Bone	1.00 ± 0.18	1.24 ± 0.44	1.18 ± 0.005	1.04 ± 0.27	1.20 ± 0.34
Brain	0.17 ± 0.014	0.14 ± 0.027	0.10 ± 0.013	0.10 ± 0.011	0.05 ± 0.005

**Table 2:** HPLC gradient elution profile for the chromatographic purification of ^18^F–C_8_–6–I radiotracer. Mobile Phase A—0.1% (*v/v*) formic acid in water and mobile phase B—0.1% (*v/v*) formic acid in acetonitrile.

Time (min)	Flow Rate (mL/min)	Mobile Phase A (%)	Mobile Phase B (%)
0.00	5	90	10
3.00	5	90	10
7.00	5	60	40
10.00	5	40	60
15.00	5	10	90
17.00	5	10	90
18.00	5	90	10
21.00	5	90	10

## Data Availability

All the data generated or analyzed during this study are included in this manuscript. Original raw data are available from the University of Saskatchewan (Department of Surgery and Department of Chemistry) and can be readily furnished upon request.

## Supplementary Materials

The supporting information can be downloaded at: https://www.mdpi.com/article/10.3390/ijms25179386/s1.

## Abbreviations

A1R: adenosine A1 receptor. α-syn: Alpha-Synuclein. C_8_–6–I: caffeine-indan dimer linked with a 6-carbon chain. CPA: N^6^-Cyclopentyladenosine. FJC: Fluoro-Jade C. TH: Tyrosine hydroxylase. Thio-S: Thioflavin S.

## References

[1] Stockwell J., Jakova E., Cayabyab F.S. (2017). Adenosine A1 and A2A receptors in the brain: Current research and their role in neurodegeneration. Molecules.

[2] Chang C.-P., Wu K.-C., Lin C.-Y., Chern Y. (2021). Emerging roles of dysregulated adenosine homeostasis in brain disorders with a specific focus on neurodegenerative diseases. J. Biomed. Sci..

[3] Jakova E., Moutaoufik M.T., Lee J.S., Babu M., Cayabyab F.S. (2022). Adenosine A1 receptor ligands bind to α-synuclein: Implications for α-synuclein misfolding and α-synucleinopathy in Parkinson’s disease. Transl. Neurodegener..

[4] Lv Y.-C., Gao A.-B., Yang J., Zhong L.-Y., Jia B., Ouyang S.-H., Gui L., Peng T.-H., Sun S., Cayabyab F.S. (2020). Long-term adenosine A1 receptor activation-induced sortilin expression promotes α-synuclein upregulation in dopaminergic neurons. Neural Regen. Res..

[5] Kakish J., Lee D., Lee J.S. (2015). Drugs that bind to α-synuclein: Neuroprotective or neurotoxic?. ACS Chem. Neurosci..

[6] Jakova E., Lee J.S. (2017). Behavior of α-synuclein–drug complexes during nanopore analysis with a superimposed AC field. Electrophoresis.

[7] Tripathi R., Saber H., Chauhan V., Tripathi K., Factor S. (2018). Parkinson Disease from long term drug abuse: Meta-analysis of amphetamine/methamphetamine and Parkinson Disease (P6. 079). Neurology.

[8] Pregeljc D., Teodorescu-Perijoc D., Vianello R., Umek N., Mavri J. (2020). How important is the use of cocaine and amphetamines in the development of Parkinson disease? A computational study. Neurotox. Res..

[9] Biswas S., Bagchi A. (2023). Study of the Effects of Nicotine and Caffeine for the Treatment of Parkinson’s Disease. Appl. Biochem. Biotechnol..

[10] Kakish J., Allen K.J.H., Harkness T.A., Krol E.S., Lee J.S. (2016). Novel Dimer Compounds That Bind α-Synuclein Can Rescue Cell Growth in a Yeast Model Overexpressing α-Synuclein. A Possible Prevention Strategy for Parkinson’s Disease. ACS Chem. Neurosci..

[11] Nwabufo C.K., Aigbogun O.P., Allen K.J., Owens M.N., Lee J.S., Phenix C.P., Krol E.S. (2021). Employing in vitro metabolism to guide design of F-labelled PET probes of novel α-synuclein binding bifunctional compounds. Xenobiotica.

[12] Moutaoufik M.T., Malty R., Amin S., Zhang Q., Phanse S., Gagarinova A., Zilocchi M., Hoell L., Minic Z., Gagarinova M. (2019). Rewiring of the human mitochondrial interactome during neuronal reprogramming reveals regulators of the respirasome and neurogenesis. iScience.

[13] Chen J., Zaer S., Drori P., Zamel J., Joron K., Kalisman N., Lerner E., Dokholyan N.V. (2021). The structural heterogeneity of α-synuclein is governed by several distinct subpopulations with interconversion times slower than milliseconds. Structure.

[14] Chen J., Zaer S., Drori P., Zamel J., Joron K., Kalisman N., Lerner E., Dokholyan N.V. (2021). Additional simulation data of α-synuclein monomer. Dataset.

[15] Thanos P.K., Wang G.-J., Volkow N.D. (2008). Positron emission tomography as a tool for studying alcohol abuse. Alcohol Res. Health.

[16] Halder R., Ritter T. (2021). 18F-Fluorination: Challenge and Opportunity for Organic Chemists. J. Org. Chem..

[17] Matthews P.M., Rabiner E.A., Passchier J., Gunn R.N. (2012). Positron emission tomography molecular imaging for drug development. Br. J. Clin. Pharmacol..

[18] Cherry S.R. (2001). Fundamentals of positron emission tomography and applications in preclinical drug development. J. Clin. Pharmacol..

[19] Ghosh K.K., Padmanabhan P., Yang C.-T., Ng D.C.E., Palanivel M., Mishra S., Halldin C., Gulyás B. (2022). Positron emission tomographic imaging in drug discovery. Drug Discov. Today.

[20] Suridjan I., Comley R.A., Rabiner E.A. (2019). The application of positron emission tomography (PET) imaging in CNS drug development. Brain Imaging Behav..

[21] Bergström M., Grahnen A., Långström B. (2003). Positron emission tomography microdosing: A new concept with application in tracer and early clinical drug development. Eur. J. Clin. Pharmacol..

[22] Lee C.-M., Farde L. (2006). Using positron emission tomography to facilitate CNS drug development. Trends Pharmacol. Sci..

[23] Gulyás B., Halldin C., Sóvágó J., Sandell J., Cselényi Z., Vas Á., Kiss B., Kárpáti E., Farde L. (2002). Drug distribution in man: A positron emission tomography study after oral administration of the labelled neuroprotective drug vinpocetine. Eur. J. Nucl. Med. Mol. Imaging.

[24] Miyoshi S., Mitsuoka K., Nishimura S., Veltkamp S.A. (2011). Radioisotopes in drug research and development: Focus on positron emission tomography. Radioisotopes—Applications in Bio-Medical Science.

[25] Sekar S., Zhang Y., Miranzadeh Mahabadi H., Buettner B., Taghibiglou C. (2023). Low-Field Magnetic Stimulation Alleviates MPTP-Induced Alterations in Motor Function and Dopaminergic Neurons in Male Mice. Int. J. Mol. Sci..

[26] Sekar S., Viswas R.S., Miranzadeh Mahabadi H., Alizadeh E., Fonge H., Taghibiglou C. (2021). Concussion/mild traumatic brain injury (TBI) induces brain insulin resistance: A positron emission tomography (PET) scanning study. Int. J. Mol. Sci..

[27] Kakish J., Tavassoly O., Lee J.S. (2014). Rasagiline, a suicide inhibitor of monoamine oxidases, binds reversibly to α-synuclein. ACS Chem. Neurosci..

[28] Draper-Joyce C.J., Khoshouei M., Thal D.M., Liang Y.-L., Nguyen A.T., Furness S.G., Venugopal H., Baltos J.-A., Plitzko J.M., Danev R. (2018). Structure of the adenosine-bound human adenosine A1 receptor–Gi complex. Nature.

[29] Ijzerman A.P., van Galen P.J., Jacobson K.A. (1992). Molecular modeling of adenosine receptors. I. The ligand binding site on the A1 receptor. Drug Des. Discov..

[30] Carpenter B., Lebon G. (2017). Human adenosine A2A receptor: Molecular mechanism of ligand binding and activation. Front. Pharmacol..

[31] Aigbogun O.P., Nwabufo C.K., Owens M.N., Allen K.J.H., Lee J.S., Phenix C.P., Krol E.S. (2022). An HPLC-UV validated bioanalytical method for measurement of in vitro Phase 1 kinetics of α-synuclein binding bifunctional compounds. Xenobiotica.

[32] Postuma R.B., Lang A.E., Munhoz R.P., Charland K., Pelletier A., Moscovich M., Filla L., Zanatta D., Rios Romenets S., Altman R. (2012). Caffeine for treatment of Parkinson disease. Neurology.

[33] Postuma R.B., Anang J., Pelletier A., Joseph L., Moscovich M., Grimes D., Furtado S., Munhoz R.P., Appel-Cresswell S., Moro A. (2017). Caffeine as symptomatic treatment for Parkinson disease (Café-PD): A randomized trial. Neurology.

[34] Prediger R.D. (2010). Effects of caffeine in Parkinson’s disease: From neuroprotection to the management of motor and non-motor symptoms. J. Alzheimer’s Dis..

[35] Hernán M.A., Zhang S.M., Rueda-DeCastro A.M., Colditz G.A., Speizer F.E., Ascherio A. (2001). Cigarette smoking and the incidence of Parkinson’s disease in two prospective studies. Ann. Neurol..

[36] Checkoway H., Powers K., Smith-Weller T., Franklin G.M., Longstreth Jr W., Swanson P.D. (2002). Parkinson’s disease risks associated with cigarette smoking, alcohol consumption, and caffeine intake. Am. J. Epidemiol..

[37] Quik M., Perez X.A., Bordia T. (2012). Nicotine as a potential neuroprotective agent for Parkinson’s disease. Mov. Disord..

[38] Biswas S., Roy R., Biswas R., Bagchi A. (2020). Structural analysis of the effects of mutations in Ubl domain of Parkin leading to Parkinson’s disease. Gene.

[39] Levin L., Srour S., Gartner J., Kapitansky O., Qutob N., Dror S., Golan T., Dayan R., Brener R., Ziv T. (2016). Parkin somatic mutations link melanoma and Parkinson’s disease. J. Genet. Genom..

[40] Wahlqvist M.L., Lee M.-S., Hsu C.-C., Chuang S.-Y., Lee J.-T., Tsai H.-N. (2012). Metformin-inclusive sulfonylurea therapy reduces the risk of Parkinson’s disease occurring with Type 2 diabetes in a Taiwanese population cohort. Park. Relat. Disord..

[41] Paudel Y.N., Angelopoulou E., Piperi C., Shaikh M.F., Othman I. (2020). Emerging neuroprotective effect of metformin in Parkinson’s disease: A molecular crosstalk. Pharmacol. Res..

[42] Agostini F., Masato A., Bubacco L., Bisaglia M. (2022). Metformin repurposing for Parkinson disease therapy: Opportunities and challenges. Int. J. Mol. Sci..

[43] Mor D.E., Sohrabi S., Kaletsky R., Keyes W., Tartici A., Kalia V., Miller G.W., Murphy C.T. (2020). Metformin rescues Parkinson’s disease phenotypes caused by hyperactive mitochondria. Proc. Natl. Acad. Sci. USA.

[44] Winklhofer K.F., Haass C. (2010). Mitochondrial dysfunction in Parkinson’s disease. Biochim. Biophys. Acta BBA-Mol. Basis Dis..

[45] Abou-Sleiman P.M., Muqit M.M., Wood N.W. (2006). Expanding insights of mitochondrial dysfunction in Parkinson’s disease. Nat. Rev. Neurosci..

[46] Malpartida A.B., Williamson M., Narendra D.P., Wade-Martins R., Ryan B.J. (2021). Mitochondrial dysfunction and mitophagy in Parkinson’s disease: From mechanism to therapy. Trends Biochem. Sci..

[47] Dimpfel W., Hoffmann J. (2011). Effects of rasagiline, its metabolite aminoindan and selegiline on glutamate receptor mediated signalling in the rat hippocampus slice in vitro. BMC Pharmacol..

[48] Chau K., Cooper J., Schapira A. (2010). Rasagiline protects against alpha-synuclein induced sensitivity to oxidative stress in dopaminergic cells. Neurochem. Int..

[49] Bar-Am O., Weinreb O., Amit T., Youdim M.B. (2010). The neuroprotective mechanism of 1-(R)-aminoindan, the major metabolite of the anti-parkinsonian drug rasagiline. J. Neurochem..

[50] Neyroud D., Cheng A.J., Donnelly C., Bourdillon N., Gassner A.-L., Geiser L., Rudaz S., Kayser B., Westerblad H., Place N. (2019). Toxic doses of caffeine are needed to increase skeletal muscle contractility. Am. J. Physiol.-Cell Physiol..

[51] Grgic J. (2021). Effects of Caffeine on Resistance Exercise: A Review of Recent Research. Sports Med..

[52] Kang J.H., Lee S.K. (2021). Progressive nicotine poisoning by multiple transdermal nicotine patches. J. Med. Life Sci..

[53] Dworzański W., Opielak G., Burdan F. (2009). Side effects of caffeine. Pol. Merkur. Lek. Organ Pol. Tow. Lek..

[54] Carstens E., Carstens M.I. (2022). Sensory effects of nicotine and tobacco. Nicotine Tob. Res..

[55] Fiore M.C., Jorenby D.E., Baker T.B., Kenford S.L. (1992). Tobacco dependence and the nicotine patch: Clinical guidelines for effective use. JAMA.

[56] Nwabufo C.K., Aigbogun O.P. (2022). Diagnostic and therapeutic agents that target alpha-synuclein in Parkinson’s disease. J. Neurol..

[57] Taylor E.M. (2002). The impact of efflux transporters in the brain on the development of drugs for CNS disorders. Clin. Pharmacokinet..

[58] Wager T.T., Hou X., Verhoest P.R., Villalobos A. (2016). Central Nervous System Multiparameter Optimization Desirability: Application in Drug Discovery. ACS Chem. Neurosci..

[59] Zhang L., Villalobos A., Beck E.M., Bocan T., Chappie T.A., Chen L., Grimwood S., Heck S.D., Helal C.J., Hou X. (2013). Design and selection parameters to accelerate the discovery of novel central nervous system positron emission tomography (PET) ligands and their application in the development of a novel phosphodiesterase 2A PET ligand. J. Med. Chem..

[60] Zhang L., Chen L., Beck E.M., Chappie T.A., Coelho R.V., Doran S.D., Fan K.H., Helal C.J., Humphrey J.M., Hughes Z. (2017). The Discovery of a Novel Phosphodiesterase (PDE) 4B-Preferring Radioligand for Positron Emission Tomography (PET) Imaging. J. Med. Chem..

[61] Kaide S., Watanabe H., Iikuni S., Hasegawa M., Ono M. (2022). Synthesis and Evaluation of 18F-Labeled Chalcone Analogue for Detection of α-Synuclein Aggregates in the Brain Using the Mouse Model. ACS Chem. Neurosci..

[62] Lindberg A., Knight A.C., Sohn D., Rakos L., Tong J., Radelet A., Mason N.S., Stehouwer J.S., Lopresti B.J., Klunk W.E. (2021). Radiosynthesis, in Vitro and in Vivo Evaluation of [18F]CBD-2115 as a First-in-Class Radiotracer for Imaging 4R-Tauopathies. ACS Chem. Neurosci..

[63] Murrell E., Tong J., Smil D., Kiyota T., Aman A.M., Isaac M.B., Watson I.D.G., Vasdev N. (2021). Leveraging Open Science Drug Development for PET: Preliminary Neuroimaging of 11C-Labeled ALK2 Inhibitors. ACS Med. Chem. Lett..

[64] Urbina F., Zorn K.M., Brunner D., Ekins S. (2021). Comparing the Pfizer Central Nervous System Multiparameter Optimization Calculator and a BBB Machine Learning Model. ACS Chem. Neurosci..

[65] Klann E.M., Dissanayake U., Gurrala A., Farrer M., Shukla A.W., Ramirez-Zamora A., Mai V., Vedam-Mai V. (2022). The Gut–Brain Axis and Its Relation to Parkinson’s Disease: A Review. Front. Aging Neurosci..

[66] Tan A.H., Lim S.Y., Lang A.E. (2022). The microbiome–gut–brain axis in Parkinson disease—From basic research to the clinic. Nat. Rev. Neurol..

[67] Chan D.G., Ventura K., Villeneuve A., Du Bois P., Holahan M.R. (2022). Exploring the Connection between the Gut Microbiome and Parkinson’s Disease Symptom Progression and Pathology: Implications for Supplementary Treatment Options. J. Park. Dis..

[68] Nguyen A.T.N., Tran Q.L., Baltos J.-A., McNeill S.M., Nguyen D.T.N., May L.T. (2023). Small molecule allosteric modulation of the adenosine A1 receptor. Front. Endocrinol..

[69] Nguyen N.T., Nguyen T.H., Pham T.N.H., Huy N.T., Bay M.V., Pham M.Q., Nam P.C., Vu V.V., Ngo S.T. (2019). Autodock vina adopts more accurate binding poses but autodock4 forms better binding affinity. J. Chem. Inf. Model..

[70] Bruns R.F., Fergus J.H. (1990). Allosteric enhancement of adenosine A1 receptor binding and function by 2-amino-3-benzoylthiophenes. Mol. Pharmacol..

[71] Ferguson G.N., Valant C., Horne J., Figler H., Flynn B.L., Linden J., Chalmers D.K., Sexton P.M., Christopoulos A., Scammells P.J. (2008). 2-aminothienopyridazines as novel adenosine A1 receptor allosteric modulators and antagonists. J. Med. Chem..

[72] Chen Z., Xiong C., Pancyr C., Stockwell J., Walz W., Cayabyab F.S. (2014). Prolonged Adenosine A1 Receptor Activation in Hypoxia and Pial Vessel Disruption Focal Cortical Ischemia Facilitates Clathrin-Mediated AMPA Receptor Endocytosis and Long-Lasting Synaptic Inhibition in Rat Hippocampal CA3-CA1 Synapses: Differential Regulat. J. Neurosci..

[73] Cleary C., Linde J., Hiscock K., Hadas I., Belmaker R., Agam G., Flaisher-Grinberg S., Einat H. (2008). Antidepressive-like effects of rapamycin in animal models: Implications for mTOR inhibition as a new target for treatment of affective disorders. Brain Res. Bull..

[74] Flaisher-Grinberg S., Einat H. (2009). A possible utilization of the mice forced swim test for modeling manic-like increase in vigor and goal-directed behavior. J. Pharmacol. Toxicol. Methods.

[75] Zhang Y., Wang T., Zhang X., Deuther-Conrad W., Fu H., Cui M., Zhang J., Brust P., Huang Y., Jia H. (2022). Discovery and development of brain-penetrant 18F-labeled radioligands for neuroimaging of the sigma-2 receptors. Acta Pharm. Sin. B.

