# Supplementary material for: The Bifunctional Dimer Caffeine-Indan Attenuates α-Synuclein Misfolding, Neurodegeneration and Behavioral Deficits after Chronic Stimulation of Adenosine A1 Receptors
Source: Int J Mol Sci. 2024 Aug 29;25(17):9386. doi: 10.3390/ijms25179386 (PMC11395333; doi:10.3390/ijms25179386)
Supplement: Supplementary file 1 [file ijms-25-09386-s001.zip › ijms-3077668-supplementary.pdf]

The Bifunctional Dimer Caffeine-Indan Attenuates α-Synuclein Misfolding, Neurodegeneration and Behavioral Deficits after Chronic Stimulation of Adenosine A1 Receptors

Supplementary Figures:

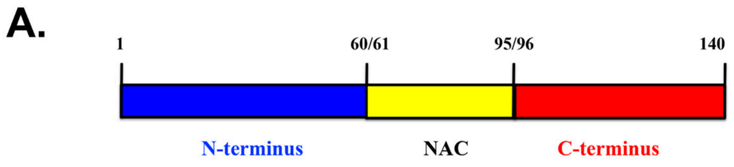

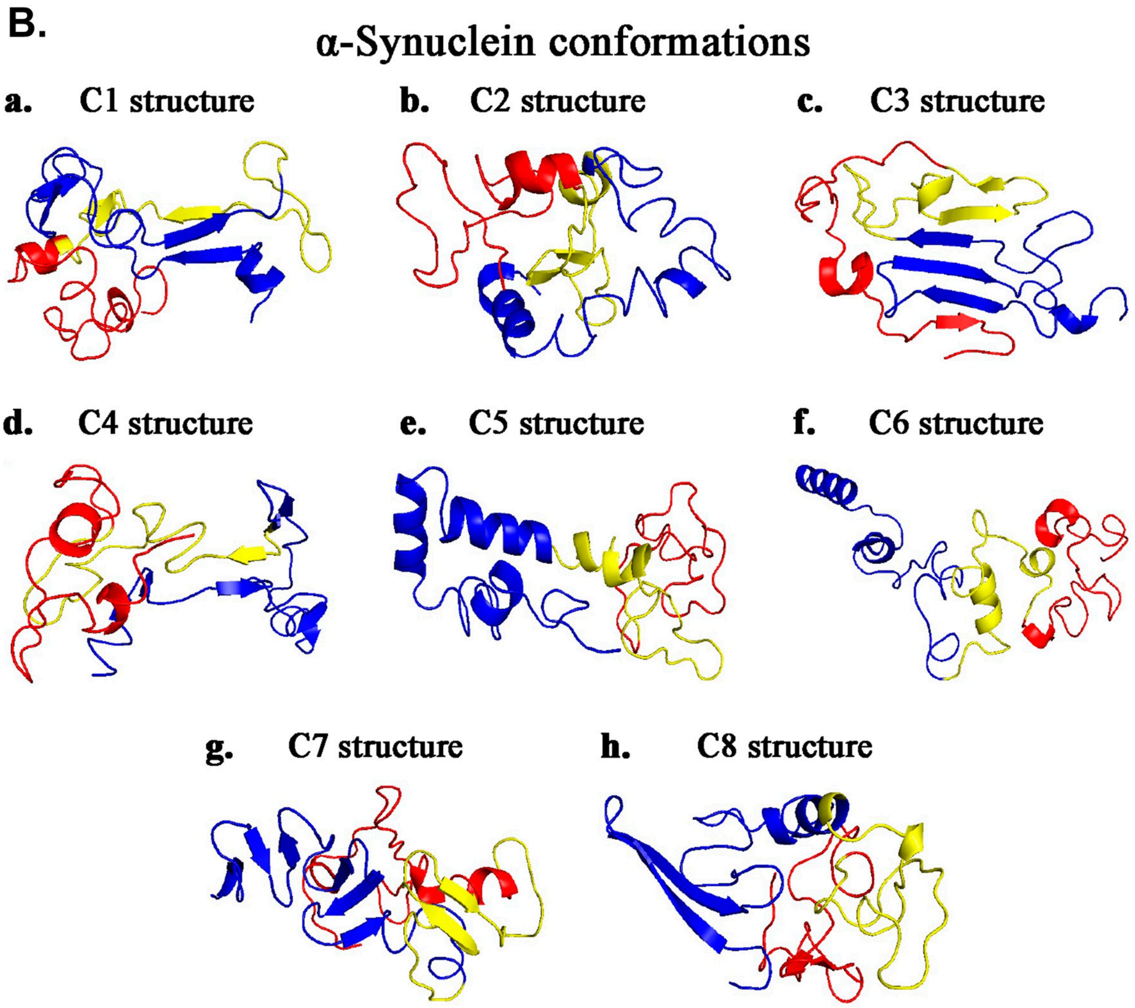

Supplementary Figure S1. A. The α-synuclein domains are denoted as follows: Blue – N-terminus; Yellow – NAC region; Red – C-terminus. **B.** Summary of the eight α-synuclein structures as

determined by discrete molecular dynamics simulations and further confirmed by far-UV circular dichroism and cross-linking mass spectrometry [13].

Supplementary Figure S2. The **a.** C4 and **b.** C6 structures show the dimer compound forming only hydrogen bonds with the caffeine moiety to C-terminus at Y133 and Y125, respectively, but they also show weak interaction with the N-terminus of the protein. In the both C4 and C6 structures, the dimer compound interacts via additional five C-terminal and one N-terminal amino acid residues. Based on this binding pattern, the C4 and C6 structures could also adopt a “loop” conformation by binding to the dimer compound.

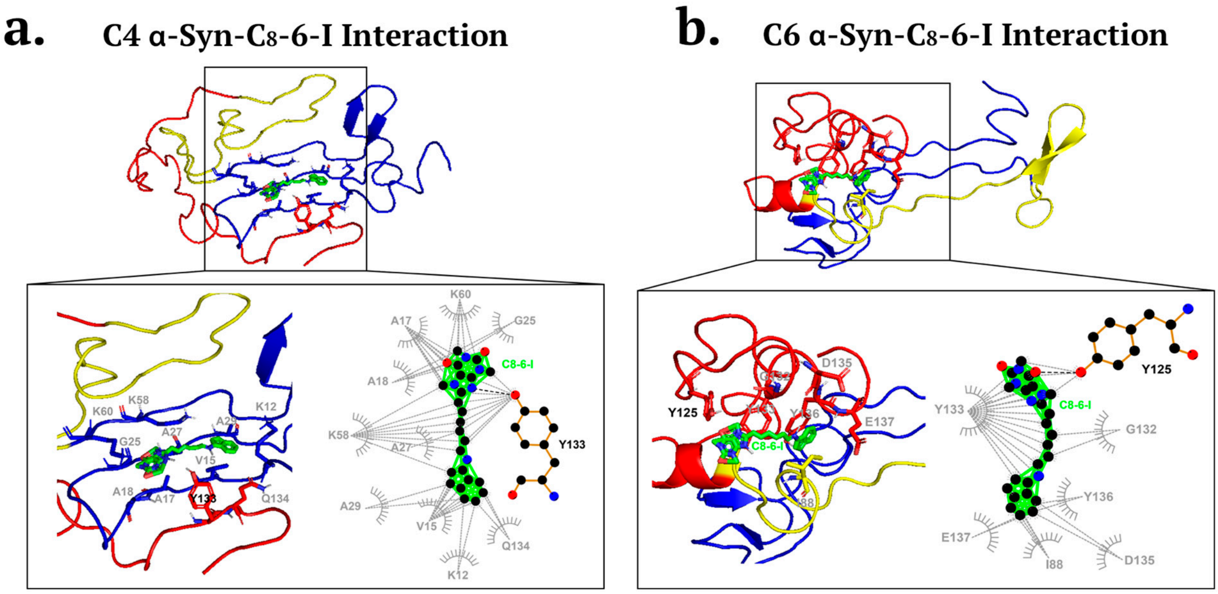

Supplementary Figure S2. Molecular docking simulation of α-Syn structures a. C4 and b. C6 bound to C_8-6-I. These structures show the dimer compound forming only hydrogen bonds with the caffeine moiety to C-terminus at Y133 and Y125, respectively, but they also show weak interaction with the N-terminus of the protein. The C4 structure surrounds the dimer compound by hydrophobic interactions with ten amino acid residues located in the C4 N-terminus (Supplementary Figure S1a). In contrast, the C6 structure interacts with the dimer compound via additional five C-terminal and one N-terminal amino acid residues. Based on this binding pattern, both C4 and C6 structures could also adopt a “loop” conformation by binding to the dimer compound.

Supplementary Figure S3 The **a.** C5 and **c.** C7 structures showed only binding to the N-terminus, which predicts a “knot” conformation which contradicts predictions from previous nanopore analysis suggesting the dimer compound adopts a “loop” conformation [10].

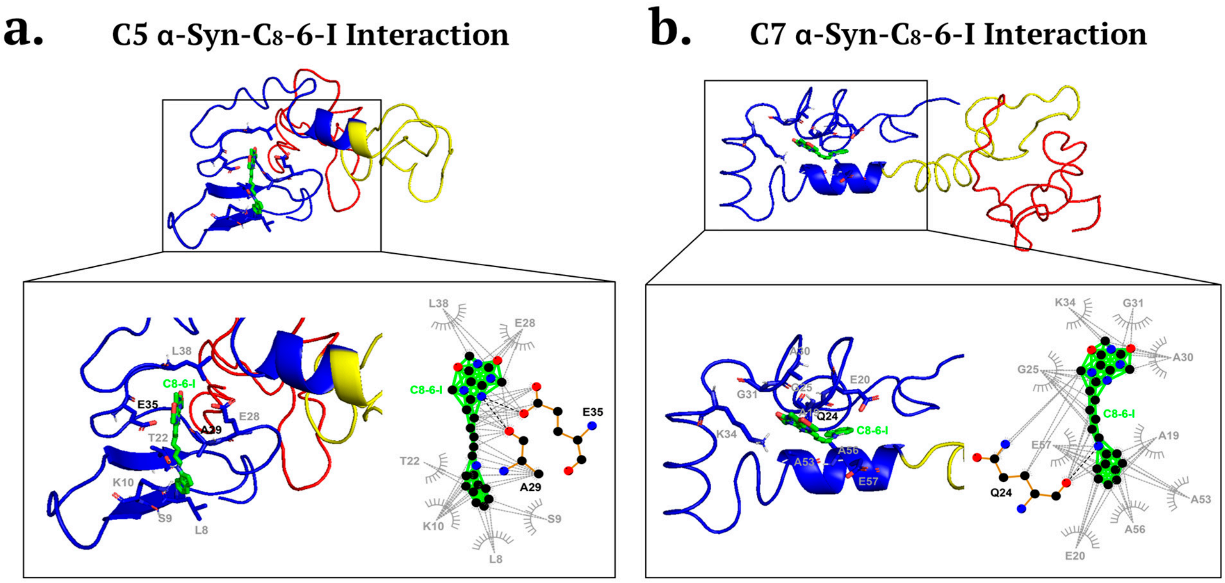

Supplementary Figure S3. Molecular docking simulation of α-Syn structures a. C5 and b. C7 bound to C_8-6-I. These structures showed only binding to the N-terminus, which predicts a "knot" conformation which contradicts predictions from previous nanopore analysis suggesting the dimer compound adopts a "loop" conformation [5].

Since we recently showed that 1-aminoindan resembles the neuroprotective effects of the A1R antagonist DPCPX in our alpha-synucleinopathy rat model [3], we tested whether 1-aminoindan (a component of the C_8-6-I dimer) could directly bind to adenosine A1 receptors, similar to DPCPX and C_8-6-I, to mediate this neuroprotective effect. Indeed, our current *in silico* findings also predicted that 1-aminoindan binds to A1R and occupies the orthosteric adenosine binding sites (see Supplementary Figure S4a,b). Our molecular docking also showed that the binding energies for 1-aminoindan interacting with A1R and A2AR were very similar, with the lowest binding energy determined to be -5.7 Kcal/mol (see Supplementary Table S1). The binding interactions of 1-aminoindan with A1R and A2AR are described in the Results section.

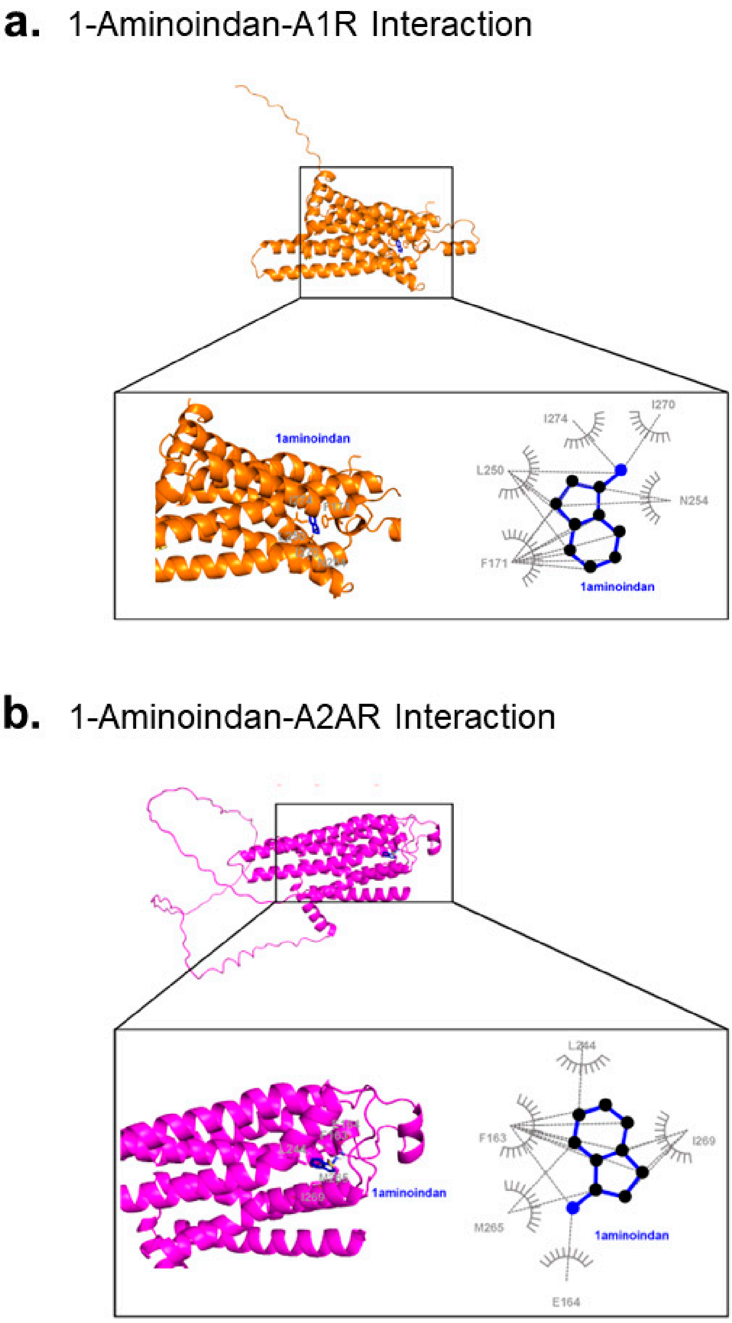

.

Supplementary Figure S4. Molecular docking of 1-aminoindan with A1R and A2AR. a. Molecular docking predicts 1-aminoindan binds to the orthosteric binding residues F171 and N254 in A1R. b. Molecular docking of 1-aminoindan with A2AR, indicating hydrophobic interaction between 1-aminoindan and A2AR; however, this binding occurs outside the orthosteric caffeine binding sites. The lowest binding energies determined from nine possible binding conformations for 1-aminoindan interacting with A1R and A2AR were very similar (i.e., lowest at -5.7 Kcal/mol, see Supplementary Table S1).

Supplementary Table S1. Binding Energies of 1-aminoindan with A1R or A2AR.

Receptor-Ligand	Binding Affinity (Kcal/mol)	RMSD Upper bound	RMSD Lower bound
A1R with 1-aminoindan	-5.7	0	0
A1R with 1-aminoindan	-5.6	3.622	2.103
A1R with 1-aminoindan	-5.5	4.122	2.146
A1R with 1-aminoindan	-5.3	3.788	1.988
A1R with 1-aminoindan	-5	31.304	29.744
A1R with 1-aminoindan	-5	6.554	5.988
A1R with 1-aminoindan	-5	31.438	29.984
A1R with 1-aminoindan	-4.9	30.968	29.767
A1R with 1-aminoindan	-4.8	45.457	44.599
A2AR with 1-aminoindan	-5.7	0	0
A2AR with 1-aminoindan	-5.4	34.316	33.085
A2AR with 1-aminoindan	-5.2	14.656	13.414
A2AR with 1-aminoindan	-5.1	34.024	32.814
A2AR with 1-aminoindan	-5.1	24.852	23.275
A2AR with 1-aminoindan	-5	26.6	24.775
A2AR with 1-aminoindan	-5	24.434	22.655
A2AR with 1-aminoindan	-4.9	34.704	33.193
A2AR with 1-aminoindan	-4.8	35.781	33.989

Our molecular docking results involving the eight alpha-synuclein structures used revealed that the binding affinities were very similar (*i.e.*, see Supplementary Table S2, the binding energies were in the range of -7.4 Kcal/mol for C5 structure to -9.8 Kcal/mol for C2 alpha-synuclein). Our molecular docking analysis also revealed that both A1R and A2AR form hydrophobic interactions with the dimer with an apparent binding energy of -5.7 Kcal/mol (see Supplementary Table S2). Notably, the dimer was predicted to bind to the agonist/antagonist orthosteric binding site F171 of A1R, whereas the dimer binding interactions did not involve the caffeine/adenosine orthosteric binding sites of A2AR (see Results section for full description).

Supplementary Table S2. Binding energies of C_8-6-I with alpha-synuclein structures and adenosine receptors (A1R and A2AR).

**C8-6-I binding to protein**	**Binding affinity (kcal/mol)**	**RMSD lower bound**	**RSMD upper bound**
**C8-6-I** binding to C1 α-Syn	-7.7	0.0	0.0
**C8-6-I** binding to C2 α-Syn	-9.8	0.0	0.0
**C8-6-I** binding to C3 α-Syn	-7.6	0.0	0.0
**C8-6-I** binding to C4 α-Syn	-7.8	0.0	0.0
**C8-6-I** binding to C5 α-Syn	-7.4	0.0	0.0
**C8-6-I** binding to C6 α-Syn	-7.9	0.0	0.0
**C8-6-I** binding to C7 α-Syn	-8.0	0.0	0.0
**C8-6-I** binding to C8 α-Syn	-8.0	0.0	0.0
**C8-6-I** binding to A1R	-5.7	0	0
**C8-6-I** binding to A2AR	-5.7	0	0
